# Selective Targeting of Immune Checkpoints HLA‐G and CD47 Using Novel Dual Signaling Protein DSP216 Promotes Innate Anticancer Immunity

**DOI:** 10.1002/advs.202521448

**Published:** 2026-02-09

**Authors:** Lisa J. Jacob, Liat Tamir, Mufeed Abdeen, Ami Tamir, Alexandra Aronin, Itai Bloch, Roy Kahn, Iris Pecker, Mark Tykocinski, Gerwin Huls, Yaron Pereg, Ayelet Chajut, Edwin Bremer

**Affiliations:** ^1^ Department of Hematology University Medical Center Groningen University of Groningen Groningen the Netherlands; ^2^ Kahr Medical Ltd Modi'in Israel; ^3^ Migal – Galilee Research Institute Kiryat‐Shmona Israel; ^4^ Department of Pathology and Genomic Medicine Sidney Kimmel Medical College Thomas Jefferson University Philadelphia Pennsylvania USA

**Keywords:** CD47, HLA‐G, immune checkpoints, immunotherapy, LILRB1, LILRB2, SIRPα

## Abstract

Immunotherapy has significantly improved treatment outcomes for cancer patients within the past decade, with breakthrough results using immune checkpoint inhibitors (ICIs), most notably those targeting the PD‐1/PD‐L1 inhibitory axis. Nevertheless, many patients and tumor types do not respond to current ICIs, and next‐generation drugs are urgently needed. Dual Signaling Protein 216 (DSP216) is a new ligand‐based immunotherapeutic—a dual HLA‐G and CD47 ICI. DSP216 was designed to exclusively bind to cells co‐expressing the immune checkpoints CD47 and HLA‐G, thereby mitigating ICI activity towards normal cells expressing only CD47 or HLA‐G and associated side effects. Computational chemistry was used to optimize DSP216 affinity to HLA‐G with the aim to achieve the desired binding mode and DSP216 binding to CD47^+^/HLA‐G^+^ and CD47^+^/HLA‐G^−^ cancer cells, PBMCs, and RBCs was tested. Functional blocking of the CD47 and HLA‐G axis was investigated in macrophage polarization and phagocytosis assays and NK cytotoxicity assays. DSP216 with an ‘active’ (DSP216a), but not with an ‘inactive’ Fc (DSP216i), triggered CD16‐signaling in a reporter cell line, and the combination of checkpoint blockade and ADCC by DSP216a potentiated NK‐mediated cytotoxicity. These encouraging findings support the continued preclinical evaluation of DSP216.

## Graphical abstract

The inhibitory immune checkpoints HLA‐G and CD47 are expressed on certain tumor types and inhibit immune cells in the tumor microenvironment. DSP216 binds specifically to cancer cells expressing both HLA‐G and CD47, and blocks their inhibitory signaling. This binding pattern prevents toxicity due to on‐target off‐tumor binding and enables (re‐) activation of anti‐cancer immunity restricted to the tumor microenvironment.

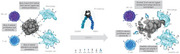

## 1 Introduction

Immunotherapy using immune checkpoint inhibitors (ICIs) has significantly improved treatment outcome for cancer patients. Nevertheless, many patients and tumor types do not respond to current ICIs, and next‐generation immune checkpoint (IC)‐directed drugs are urgently needed. One approach to this end is the development of a dual signaling protein (DSP) platform, wherein the extracellular domains (ECDs) of each of two proteins are fused together to create unique immunotherapeutic functionality through coordinated receptor‐ligand interactions. This is exemplified by DSP107, a paradigmatic DSP in which the ECDs of signal regulatory protein α (SIRPα) and 4‐1BB ligand (4‐1BBL) are fused, creating a molecule that simultaneously acts as a checkpoint inhibitor and T cell stimulator. [1] DSP107 is currently being evaluated in clinical trials for the treatment of hematological malignancies and solid tumors. [2]

Simultaneous targeting of the ICs human leukocyte antigen G (HLA‐G) and CD47 via a DSP approach represents another interesting option. HLA‐G, a pivotal regulator of immune tolerance during pregnancy, is aberrantly upregulated in many cancer types, like glioma, thyroid cancer, breast cancer, oral squamous cell carcinoma, gastritic cancer, esophageal cancer, liver cancer, lung cancer, colorectal cancer, pancreatic cancer, cervical cancer, and ovarian cancer. [3] High expression of HLA‐G correlates with shorter survival and is an independent prognostic risk factor for cancer patients. [3]

HLA‐G binds to the inhibitory receptors leukocyte immunoglobulin like receptor B1 (LILRB1) and leukocyte immunoglobulin like receptor B2 (LILRB2) on macrophages, neutrophils, dendritic, T, NK, and B cells—thereby, HLA‐G suppresses both innate and adaptive anti‐cancer immunity in a multi‐fold manner. [3] For instance, HLA‐G restrains NK cytotoxicity, IFN‐γ production, and trans‐endothelial migration, and it negatively impacts T cell proliferation, cytotoxicity, chemotaxis, and CD4 T cell responsiveness. [3] Further, HLA‐G triggers polarization of macrophages toward a pro‐tumoral (M2) phenotype and inhibits B cell proliferation, differentiation and antibody secretion. [3] Targeting of HLA‐G can reverse immune inhibition specifically in the tumor microenvironment, as HLA‐G is almost exclusively expressed in the tumor microenvironment, with HLA‐G expression on healthy tissues being very restricted. [4, 5, 6]

Notably, despite its role in cancer having been discovered over 25 years ago [7] and it being an immune checkpoint and cancer‐specific target, HLA‐G has so far not been successfully targeted in a clinical setting, with only one HLA‐G antibody currently being evaluated in a clinical trial. [8, 9] Indeed, antibody‐based targeting of HLA‐G has proven challenging, hampered, in part, by the existence of multiple HLA‐G isoforms. [3] Four membrane‐bound (HLA‐G1–4) and three soluble (HLA‐G5–7) HLA‐G isoforms have been reported to date. These isoforms differ in the presence of a transmembrane domain, number of extracellular immunoglobulin‐like domains, and association with non‐covalently bound beta‐2‐microglobulin (β2m). [3]

As an alternative to antibody‐based targeting, a ligand‐based approach, using the ECD of the HLA‐G receptor LILRB2, can neutralize the effects of multiple HLA‐G isoforms at once, namely HLA‐G1, HLA‐G2, HLA‐G5, and HLA‐G6. [10] Since the other HLA‐G receptor, LILRB1, binds to only the β2m‐associated isoforms HLA‐G1 and HLA‐G5, it is an inferior choice for ligand‐based HLA‐G targeting. In addition, LILRB2 has the further advantage that it may also sterically interfere with LILRB1/HLA‐G binding [10] and other potential HLA‐G binding partners, thus affording, in principle, a broadly neutralizing therapy.

The second IC, CD47, is a well‐established immune checkpoint that primarily inhibits innate immunity by activating SIRPα immune inhibitory signaling on myeloid effector cells. [11, 12] CD47 is overexpressed on nearly all cancer types [13], which makes HLA‐G the restricting factor for simultaneous targeting of HLA‐G and CD47, with HLA‐G being upregulated on glioma, thyroid cancer, breast cancer, oral squamous cell carcinoma, gastritic cancer, esophageal cancer, liver cancer, lung cancer, colorectal cancer, pancreatic cancer, cervical cancer and ovarian cancer as stated above. [3] A recent publication offers a tool to visualize co‐expression per cancer type. [14] CD47 represents the prototype ‘don't eat me’ signal toward various phagocytes, such as dendritic cells, macrophages, and granulocytes. Blocking CD47 binding to SIRPα on phagocytes potentiated cancer cell phagocytosis and enhanced innate immunity in pre‐clinical studies. [1, 15, 16] Moreover, in murine models, CD47 blockade also triggered antigen presentation and protective anti‐cancer T cell immunity. [17]

A number of CD47 targeting agents are in clinical development. While the CD47 blocking antibody magrolimab showed early promise for the treatment of hematological malignancies [18, 19] in a Phase III study, development was halted due to increased adverse events and incidence of death in the treatment arm. [11] Of note, a Phase I trial with ALX‐148, which offers a ligand‐based CD47‐targeting approach that deploys a silenced IgG1 Fc domain, has demonstrated safety and efficacy. [21]

Here, we report on the preclinical characterization of a novel therapeutic fusion protein that co‐targets HLA‐G and CD47. Specifically, dual signaling protein 216 (DSP216) comprises the ECDs of SIRPα and LILRB2, each fused alternatively to either an active or silent human IgG1. Heterodimerization is driven by knob‐in‐hole mutations in the Fc domain. The sequence of the LILRB2 fusion protein was optimized using computational modelling to achieve stronger binding to HLA‐G. Coupling the SIRPα domain to the designed LILRB2 domain yielded AND‐gate binding behavior, with minimal binding to cells that express CD47 only (peripheral blood mononuclear cells (PBMCs); red blood cells (RBCs)), but strong binding due to enhanced avidity, along with ICI activity, in the case of cells that co‐express CD47 and HLA‐G.

## 2 Results

### 2.1 DSP216 Optimization by Enhancing Affinity for HLA‐G

**FIGURE 1:**
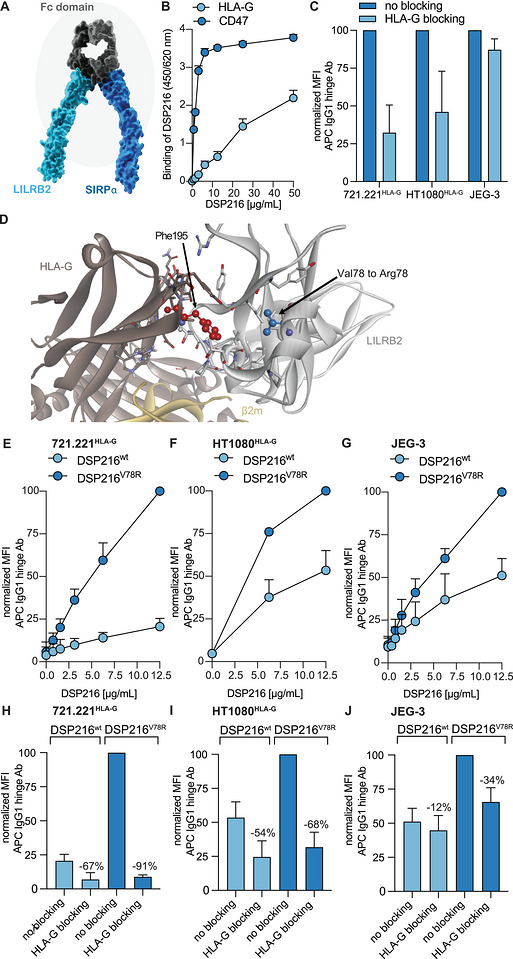
DSP216 structure and depiction of LILRB2 mutation (V78R) to enhance DSP216 binding to HLA‐G. (A) 3D schematic representation of heterodimeric DSP216 with a LILRB2 binding domain (light blue) fused to an Fc domain (dark gray) and a SIRPα binding domain (dark blue) fused to an Fc domain (light gray). (B) DSP216 binding to HLA‐G‐his and CD47‐Fc was analyzed by ELISA (*n* = 3, technical replicates, mean + standard deviation). (C) Binding of 12.5 µg/mL DSP216^wt^ without blocking (dark blue bars) and with HLA‐G blocking (light blue bars) to 721.221^HLA‐G^ cells, HT1080^HLA‐G^ cells and JEG‐3 cells detected by flow cytometry using APC‐IgG1 hinge mAb (Binding data was normalized per experiment with 100% defined as DSP216^wt^ binding without HLA‐G blocking, *n* = 3, experimental replicates, mean + standard deviation). (D) Depiction of the binding interface between LILRB2 and HLA‐G generated using the PDB structure 2DYP. In the interaction interface between HLA‐G and wild‐type LILRB2, only a single dominant difference exists between HLA‐G and its homologues: Phe195. Substituting Val78 of LILRB2 with Arg did increase the binding of DSP216 to HLA‐G^+^ CD47^+^ cells. (E/F/G) Binding of DSP216^wt^ (light blue symbols) and DSP216^V78R^ (dark blue symbols) to (E) 721.221^HLA‐G^ cells (F) HT1080^HLA‐G^ cells and (G) JEG‐3 cells was detected as in (C) (Binding data was normalized with 100% defined as DSP216^V78R^ binding without HLA‐G blocking, *n* = 3, experimental replicates, mean + standard deviation). (H/I/J) Binding of 12.5 µg/mL DSP216^wt^ (light blue bars) and DSP216^V78R^ (dark blue bars) to (H) 721.221^HLA‐G^ cells (I) HT1080^HLA‐G^ cells, and (J) JEG‐3 cells with or without HLA‐G blocking (*n* = 3, experimental replicates, mean + standard deviation).

DSP216 comprises LILRB2 and SIRPα ECDs, each fused to a heterodimerizing IgG1 Fc (Figure 1A), designed to coordinately block inhibitory signaling by HLA‐G and CD47 co‐expressed on cancer cells. A common off‐tumor effect of Ab‐based CD47 blocking agents is anemia, attributed to their binding CD47 on RBCs and effecting hemolysis. [11] To avoid this side effect, DSP216 is designed to selectively bind and effectively trigger inhibitory checkpoint signaling on HLA‐G and CD47 positive cells due to high(er) avidity upon dual binding. Conversely, DSP216 is designed to have no/minimal ICI effects on cells expressing CD47, but not HLA‐G, such as normal PBMCs and RBCs. A prototype DSP216 with an IgG4 Fc indeed bound dose‐dependently to HLA‐G and CD47 in ELISA (Figure 1B) and a prototype DSP216 with IgG1 Fc, designated wild type (DSP216^wt^) indeed bound to the three HLA‐G and CD47 positive cell lines—721.221^HLA‐G^, HT1080^HLA‐G^ and JEG‐3 (Figure 1C; for cell surface expression levels of HLA‐G and CD47 on cell lines see Figure S1; for gating strategies and for representative flow cytometry plots, see Figures S2–S4). Notably, binding of DSP216^wt^ to HLA‐G transduced cells was only slightly higher than to empty vector transduced or wild‐type cells (Figure S5A,C). Further pre‐incubation with HLA‐G blocking antibody only marginally inhibited binding of DSP216^wt^ (Figure 1C), especially to the JEG‐3 cell line with endogenous HLA‐G expression, suggesting that the SIRPα domain was the main driver of the interaction.

To increase the contribution of LILRB2‐mediated interactions to DSP216 binding and thus increase overall binding of DSP216 to HLA‐G and CD47 co‐expressing cells, computational modelling was performed to identify amino acid substitutions in the LILRB2 domain with a predicted positive impact on LILRB2 binding to HLA‐G (Table S1). In line with the computational modeling, pre‐incubation with HLA‐G blocking mAb more strongly impacted binding of these DSP216 variants, as compared to DSP216^wt^, to HLA‐G and CD47 double‐positive cells (Table S2). Based on this initial flow cytometric screening, one of the mutational variants, DSP216^V78R^, was selected for further investigation. The binding of this variant to HLA‐G transduced cells was considerably higher than to empty vector transduced or wild‐type cells (Figure S5B,D). The structural analysis of the binding interface between HLA‐G and LILRB2 (based on PDB ID 2DYP) and the place of the substitution (Val78 to Arg78) are depicted in Figure 1D. In line with this modeling, DSP216^V78R^ binding to 721.221^HLA‐G^ cells increased approximately 5‐fold, and approximately 2‐fold to HT1080^HLA‐G^ and JEG‐3 cells (Figure 1E–G; dark blue symbols vs. light blue symbols).

As noted above, pre‐incubation with HLA‐G blocking antibody reduced the binding of DSP216^wt^ to 721.221^HLA‐G^, HT1080^HLA‐G,^ and JEG‐3 cells only marginally, with a stronger blocking‐effect observed for DSP216^V78R^ (Figure 1H–J; light blue bars vs. dark blue bars). More specifically, for DSP216^V78R^, HLA‐G blocking antibody reduced binding to 721.221^HLA‐G^ cells by 91% (vs. 67% with DSP216^wt^), to HT1080^HLA‐G^ cells by 68% (vs. 54% with DSP216^wt^), and to JEG‐3 cells by 34% (vs. 12% with DSP216^wt^). Thus, the V78R mutation in the LILRB2 binding domain indeed increases DSP216 binding to double‐positive cells by enhancing the LILRB2/HLA‐G interaction. Based on this data, DSP216^V78R^, hereafter referred to as DSP216, was selected for further preclinical development.

### 2.2 DSP216 Binds Primarily to HLA‐G^+^ CD47^+^ Cancer Cells and Minimally to Red Blood Cells

**FIGURE 2:**
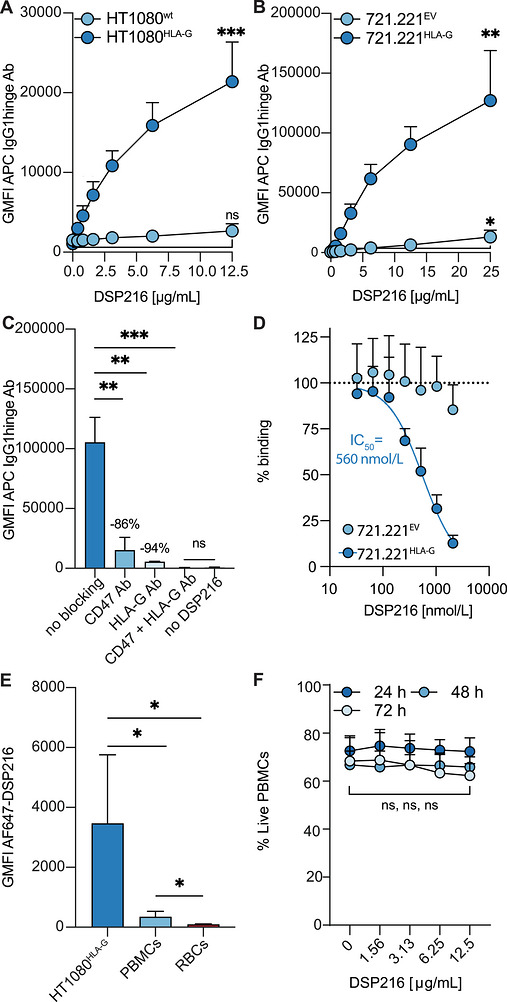
DSP216 binding, toxicity, and competition with SIRPα (A) Binding of DSP216 to HLA‐G^−^ CD47^+^ HT1080^wt^ cells (light blue symbols) and HLA‐G^+^ CD47^+^ HT1080^HLA‐G^ cells (dark blue symbols) detected by flow cytometry using APC‐IgG1 hinge Ab. Parametric unpaired t‐test was used to test differences in DSP216 binding (*n* = 4, experimental replicates, mean + standard deviation, HT1080^wt^ vs. HT1080^HLA‐G^ cells, 12.5 µg/mL *p* = 0.0003 ^***^; HT1080^wt^ 0 µg/mL vs. 12.5 µg/mL *p* = 0.0783 ns). (B) Binding of DSP216 to HLA‐G^−^ CD47^+^ 721.221^EV^ cells (light blue symbols) and HLA‐G^+^ CD47^+^ 721.221^HLA‐G^ cells (dark blue symbols) detected as in (A). Parametric unpaired t‐test was used to test differences in DSP216 binding (*n* = 3, experimental replicates, mean + standard deviation, 721.221^EV^ vs. 721.221^HLA‐G^ cells, 25 µg/mL *p* = 0.0094^**^; 721.221^EV^ 0 µg/mL vs. 25 µg/mL *p* = 0.0192^*^). (C) Binding of 25 µg/mL DSP216 to 721.221^HLA‐G^ cells with and without CD47 and/or HLA‐G blocking antibodies detected as in (A). Parametric unpaired t‐test was used to test differences in DSP216 binding (*n* = 3, experimental replicates, mean + standard deviation, medium vs. CD47 blocking *p* = 0.0026^**^, medium vs. HLA‐G blocking *p* = 0.00118^*^, medium vs. CD47 and HLA‐G blocking *p* = 0.0009^***^; 0 µg/mL DSP216 vs. CD47 and HLA‐G blocking *p* = 0.8766 ns). (D) Inhibition of SIRPα binding to 721.221^HLA‐G^ cells (dark blue symbols) and 721.221^EV^ cells (light blue symbols) with increasing concentrations of DSP216 detected using APC Penta‐His antibody by flow cytometry (*n* = 3, experimental replicates, mean + standard deviation, IC_50_ was calculated with GraphPad Prism Version 10.2.3, fitting to a four‐parameter logistic curve, IC_50_ for 721.221^EV^ cells was not reached). (E) Binding of 12.5 µg/mL AF647‐labelled DSP216 to HT1080^HLA‐G^ cells, PBMCs, and RBCs mixed detected by flow cytometry. Parametric unpaired t‐test was used to test differences in DSP216 binding (*n* = 4, biological replicates, mean + standard deviation, HT1080^HLA‐G^ vs. RBCs *p* = 0.0253^*^, HT1080^HLA‐G^ vs. PBMCs *p* = 0.0346^*^, PBMCs vs. RBCs *p* = 0.0280^*^). (F) Percentage of live PBMCs incubated with DSP216 for 24 h, 48 h, and 72 h (*n* = 6, biological replicates, mean + standard deviation, medium vs. 12.5 µg/mL DSP216, 24 h *p* = 0.725; 48 h *p* = 0.6423; 72 h *p* = 0.1736). ^*^ *p* < 0.05; ^**^ *p* < 0.01; ^***^ *p* < 0.001; ^****^ *p* < 0.0001.

The aim was to achieve AND‐gate binding fashion and AND‐gate ICI with DSP216. The term ‘AND‐gate’ originates from Boolean algebra and, in brief, means that an outcome occurs if all inputs are ON only. [22] DSP216 binding should follow the AND‐gate logic and only occur when both targets are present (ON). Possible AND‐gate binding for DSP216 was evaluated using CD47 and HLA‐G double‐positive and CD47 single‐positive cells. On HT1080 wild‐type (wt) cells expressing solely CD47 (Figure S1D), DSP216 binding was insignificant (Figure 2A, light blue symbols). In contrast, DSP216 dose‐dependently bound to the same cell line when ectopically expressing HLA‐G (Figure 2A, dark blue symbols), with an 8‐fold increase in signal on HT1080^HLA‐G^ cells compared to HT1080^wt^ cells. Similarly, DSP216 binding to 721.221^HLA‐G^ was significantly increased by 10‐fold compared to 721.221^EV^ cells (Figure 2B, see Figure S1F for histograms). Importantly, competitive inhibition of the CD47 interaction using a CD47 mAb significantly reduced DSP216 binding by 86% (Figure 2C). Likewise, competitive inhibition of HLA‐G interaction significantly reduced DSP216 binding by 94% (Figure 2C). Simultaneous competitive inhibition of CD47 and HLA‐G interaction completely abrogated binding (Figure 2C). Significant DSP216 binding when both targets were present only supports the presence of the intended AND‐gate binding fashion of DSP216.

In line with this interpretation, DSP216 inhibited binding of recombinant SIRPα to 721.221^HLA‐G^ CD47 and HLA‐G co‐expressing cells (Figure 2D, dark blue symbols, IC_50_ = 560 nmol/L and binding histograms Figure S6A,B). By contrast, DSP216 did not inhibit binding of SIRPα to 721.221^EV^ cells (Figure 2D, light blue symbols). Together, these data indicate that DSP216 effectively blocks the CD47 checkpoint on CD47 and HLA‐G double‐positive cells. Of note, since recombinant LILRB2 binding to 721.221^HLA‐G^ cells could not be detected, competition experiments with recombinant LILRB2 could not be performed (Figure S6C). Selective DSP216 binding was also observed in a mixed culture of HT1080^HLA‐G^ cells, PBMCs, and RBCs, where DSP216 bound significantly less to PBMCs than to HT1080^HLA‐G^ cells and significantly less to RBCs than to PBMCs and HT1080^HLA‐G^ cells (Figure 2E, for gating strategy see Figure S7, for representative flow cytometry plots see Figure S8A). Among PBMC subpopulations, DSP216 exhibited the highest binding affinity for monocytes, presumably due to the expression of the Fc receptor CD64 on monocytes, with lower binding observed in T cells and NK cells (see Figure S8B). Importantly, the weak binding by DSP216 to PBMCs had no significant effect on PBMCs viability after 24 h, 48 h, and 72 h (Figure 2F).

### 2.3 DSP216 Treatment Prevents HLA‐G‐mediated Polarization of Macrophages

**FIGURE 3:**
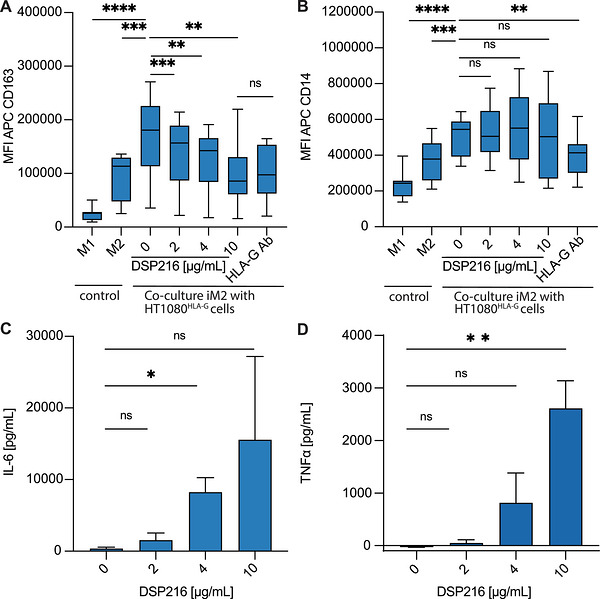
Repolarization of M2 macrophages by DSP216: Box and whiskers plots (center lines show the means, box limits indicate the 25^th^ and 75^th^ percentiles as determined by GraphPad prim 10.2.3., and whiskers extend to minimum and maximum values) depicting cell surface expression levels of polarization markers (A) CD163 and (B) CD14 on M1 macrophages (M1) and M2 macrophages (M2) (control) or immature M2 macrophages (iM2) cocultured with HT1080^HLA‐G^ cells and treated with DSP216 or HLA‐G mAb measured by flow cytometry. Parametric paired t‐test was used to test if differences in surface expression were significant (CD163 *n* = 11, biological replicates, M1 vs. iM2 p≤0.0001^****^, M2 vs. iM2 *p* = 0.0003^***^, iM2 vs. iM2 2 µg/mL DSP216 *p* = 0.0006^***^, iM2 vs. iM2 4 µg/mL DSP216 *p* = 0.0033^***^, iM2 vs. iM2 10 µg/mL DSP216 *p* = 0.0016^**^, iM2 10 µg/mL DSP216 vs. HLA‐G mAb *p* = 0.9940 ns; CD14 *n* = 9, biological replicates, M1 vs. iM2 p≤0.0001^****^, M2 vs. iM2 *p* = 0.0001^***^, iM2 vs. iM2 2 µg/mL DSP216 *p* = 0.2821 ns, iM2 vs. iM2 4 µg/mL DSP216 *p* = 0.2593 ns, iM2 vs. iM2 10 µg/mL DSP216 *p* = 0.9776 ns, iM2 vs. HLA‐G mAb *p* = 0.0017^**^). (C/D) Concentration of (C) IL‐6 and (D) TNFα in supernatant of iM2 cocultured with HT1080^HLA‐G^ cells treated with DSP216 measured by CBA. Parametric paired t‐test was used to test if differences in cytokine concentrations were significant (IL‐6 *n* = 3, biological replicates, mean + standard deviation, iM2 vs. iM2 2 µg/mL DSP216 *p* = 0.2008 ns, iM2 vs. iM2 4 µg/mL DSP216 *p* = 0.0180^*^, iM2 vs. iM2 10 µg/mL DSP216 *p* = 0.1489 ns; TNFα *n* = 3, biological replicates, mean + standard deviation, iM2 vs. iM2 2 µg/mL DSP216 *p* = 0.3132 ns, iM2 vs. iM2 4 µg/mL DSP216 *p* = 0.1319 ns, iM2 vs. iM2 10 µg/mL DSP216 *p* = 0.0132^*^). ^*^ *p* < 0.05; ^**^ *p* < 0.01; ^***^ *p* < 0.001; ^****^ *p* < 0.0001.

It has been previously reported that blocking of LILRB2 signaling can prevent polarization of macrophages toward a tumor‐promoting (M2) type characterized by CD163 expression. [23] In line with this finding, coculture of immature macrophages (iM2) with HLA‐G‐expressing cancer cells significantly upregulated surface expression levels of CD163, as compared to fully polarized M1 and M2 macrophages (Figure 3A). Importantly, CD163 is a macrophage marker commonly upregulated in M2 and tumor‐resident macrophages. [24, 25, 26] Treatment with DSP216 significantly and dose‐dependently prevented the upregulation of CD163 (Figure 3A). This inhibitory effect of DSP216 on CD163 expression was similar to that observed with HLA‐G antibody treatment (Figure 3A). In contrast, expression of CD14 was not affected by treatment with DSP216 (Figure 3B), although HLA‐G antibody did significantly reduce its expression (Figure 3B). Such loss of CD14 is detected in M2a macrophages, as compared to M1 macrophages, with CD14 being unchanged in M2c macrophages [27], and could possibly be caused by the IgG4 domain of the HLA‐G antibody. [28] This suggests that DSP216 and HLA‐G antibody may affect macrophage polarization differently. Lastly, DSP216 treatment dose‐dependently increased IL‐6 and TNFα concentrations in the supernatant of cocultures (Figures 3C,D), suggesting that DSP216 does not only affect expression of surface markers but also reprograms macrophages. In conclusion, DSP216 prevents CD163 upregulation and increases IL‐6 and TNFα secretion by macrophages cocultured with HLA‐G and CD47 co‐expressing cells.

### 2.4 DSP216 Treatment Promotes Macrophage Phagocytosis of HLA‐G^+^ CD47^+^ Cancer Cells

**FIGURE 4:**
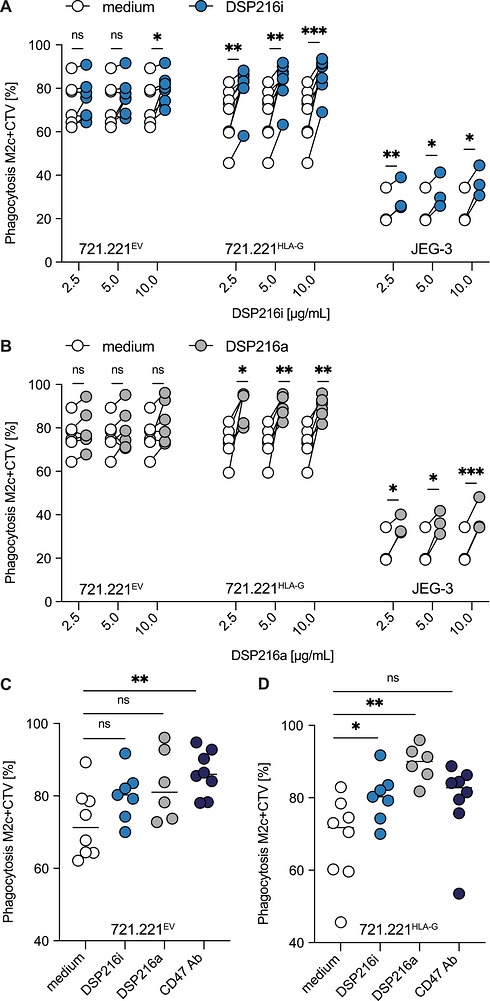
DSP216 mediated phagocytosis of CellTrace Violet (CTV) stained cancer cells. The percentage of APC‐CD11b Ab labelled macrophages positive for CTV dye is given as phagocytosis M2c+CTV [%]. (A) Phagocytosis of HLA‐G^−^ CD47^+^ 721.221^EV^ cells and HLA‐G^+^ CD47^+^ 721.221^HLA‐G^ and JEG‐3 cells by M2c macrophages alone (white symbols) or with 2.5/5/10 µg/mL DSP216 with ‘inactive’ Fc (DSP216i, blue symbols). Each line represents an individual donor (biological replicate). Parametric paired t‐test was used to test if difference between treated and untreated samples was significant (721.221^EV^: 2.5 µg/mL *p* = 0.1665 ns, 5 µg/mL *p* = 0.1075 ns, 10 µg/mL *p* = 0.0271^*^; 721.221^HLA‐G^: 2.5 µg/mL *p* = 0.0023^**^, 5 µg/mL *p* = 0.0027^**^, 10 µg/mL *p* = 0.0007^***^; JEG‐3: 2.5 µg/mL *p* = 0.0094^**^, 5 µg/mL *p* = 0.0181^*^, 10 µg/mL *p* = 0.0187^*^). (B) Phagocytosis of HLA‐G^−^ CD47^+^ 721.221^EV^ cells and HLA‐G^+^ CD47^+^ 721.221^HLA‐G^ and JEG‐3 cells by M2c macrophages alone (white symbols) or with 2.5/5/10 µg/mL DSP216 with ‘active’ Fc (DSP216a, gray symbols). Analyzed as in (A) (721.221^EV^: 2.5 µg/mL *p* = 0.0871 ns, 5 µg/mL *p* = 0.3834 ns, 10 µg/mL *p* = 0.0934 ns; 721.221^HLA‐G^: 2.5 µg/mL *p* = 0.0200^*^, 5 µg/mL *p* = 0.0075^**^, 10 µg/mL *p* = 0.0034^**^; JEG‐3: 2.5 µg/mL *p* = 0.0458^*^, 5 µg/mL *p* = 0.0427^*^, 10 µg/mL *p* = 0.0006^***^). (C) Phagocytosis of HLA‐G^−^ CD47^+^ 721.221^EV^ cells with medium (white symbols, *n* = 8, biological replicates), 10 µg/mL DSP216i (dark blue symbols, *n* = 7, biological replicates), 10 µg/mL DSP216a (gray symbols, *n* = 6, biological replicates) or 6 µg/mL mCD47 Ab (midnight blue symbols, *n* = 8, biological replicates). Parametric unpaired t‐test was used to test if differences in phagocytosis between treatments were significant (medium vs. DSP216i *p* = 0.1042 ns, medium vs. DSP216a *p* = 0.0702 ns, medium vs. mCD47 Ab *p* = 0.0041^**^). (D) Phagocytosis of HLA‐G^+^ CD47^+^ 721.221^HLA‐G^ cells with medium (white symbols, *n* = 8, biological replicates), 10 µg/mL DSP216i (dark blue symbols, *n* = 7, biological replicates), 10 µg/mL DSP216a (gray symbols, *n* = 6, biological replicates) or 6 µg/mL mCD47 Ab (midnight blue symbols, *n* = 8, biological replicates). Parametric unpaired t‐test was used to test if differences in phagocytosis between treatments were significant (medium vs. DSP216i *p* = 0.0378^*^, medium vs. DSP216a *p* = 0.0017^**^, medium vs. mCD47 Ab *p* = 0.0773 ns). ^*^ *p* < 0.05; ^**^ *p* < 0.01; ^***^ *p* < 0.001; ^****^ *p* < 0.0001.

In addition to dual checkpoint inhibition, the Fc domain of DSP216 could trigger anticancer immunity through Fc‐mediated processes such as macrophage‐mediated antibody‐dependent cellular phagocytosis (ADCP) or NK‐mediated antibody‐dependent cellular cytotoxicity (ADCC). [29] However, an active Fc domain could also introduce the risk of increased off‐tumor side effects. To this end, a DSP216 variant with a regular ‘active’ human IgG1 Fc domain (termed DSP216a) was functionally evaluated side‐by‐side with a DSP216 variant bearing an ‘inactive’ Fc (termed DSP216i) — the latter characterized by L234A L235A (LALA) mutations in the Fc backbone that abrogate binding to Fc receptors (FcR). In a phagocytosis assay, wherein macrophages are evaluated for uptake of stained cancer cells within 3 h, DSP216i dose‐dependently (Figure S10A) and significantly increased phagocytosis of CD47 and HLA‐G double‐positive 721.221^HLA‐G^ and JEG‐3 cells by macrophages (Figure 4A, for gating strategy see Figure S11 and for representative flow cytometry plots see Figure S12). By contrast, phagocytosis of CD47 single‐positive 721.221^EV^ cells with DSP216i was not significantly different from phagocytosis without DSP216 at the lower concentrations under the current experimental conditions (Figure 4A). DSP216a similarly and dose‐dependently (Figure S10B) increased phagocytosis of 721.221^HLA‐G^ and JEG‐3 cells (Figure 4B), but phagocytosis of CD47 single‐positive 721.221^EV^ cells was not significantly different than phagocytosis without DSP216a under the current experimental conditions (Figure 4B). Significant differences in phagocytosis induced by DSP216i and DSP216a were not detected for any cell line (Figure S10C–E), perhaps because maximum phagocytosis was already achieved with DSP216i.

When evaluating DSP216i, DSP216a, and CD47 mAb side‐by‐side, only CD47 mAb significantly increased phagocytosis of 721.221^EV^ cells. For DSP216i and DSP216a, no significant difference in phagocytosis was detected under the current experimental conditions (Figure 4C). Treatment of 721.221^HLA‐G^ cells with CD47 mAb did not show a statistically significant difference in phagocytosis under the current experimental conditions (Figure 4D). In line with previous results, DSP216i and DSP216a did block the CD47 IC and significantly increased phagocytosis of 721.221^HLA‐G^ cells (Figure 4D).

### 2.5 DSP216 Promotes NK Killing of HLA‐G^+^ CD47^+^ Cancer Cells

**FIGURE 5:**
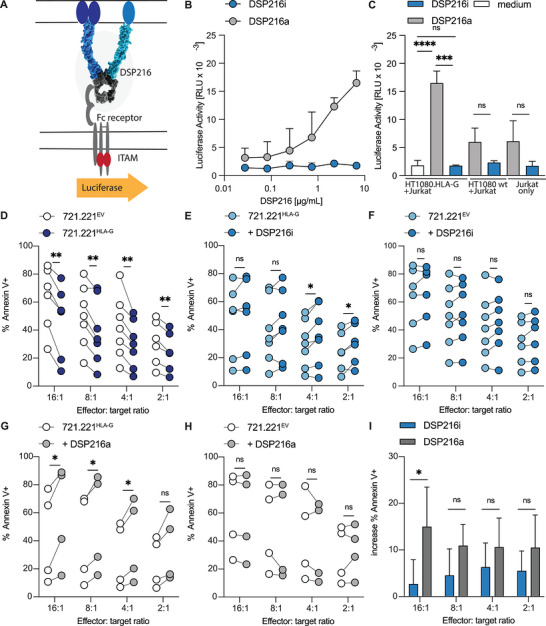
DSP216 mediated NK cytotoxicity (A) Schematic representation of luminescence‐based CD16 Fc receptor reporter assay. (B) Detected luminescence in coculture of Jurkat‐Lucia^TM^ NFAT‐CD16 reporter cell line with HT1080^HLA‐G^ cells and increasing concentrations of DSP216 with ‘active’ Fc (DSP216a, gray symbols) and DSP216 with ‘inactive’ Fc (DSP216i, blue symbols) (*n* = 3, experimental replicates, mean + standard deviation). (C) Detected luminescence in cultures of Jurkat‐Lucia^TM^ NFAT‐CD16 reporter cell line. Jurkat‐Lucia^TM^ NFAT‐CD16 cells were cocultured with HT1080^HLA‐G^ or HT1080^wt^ cells or alone and treated with medium (white bar), 6.7 µg/mL DSP216a (gray bars) or DSP216i (blue bars)(*n* = 3, experimental replicates, mean + standard deviation). Parametric unpaired t‐test was used to test if differences were significant (HT1080^HLA‐G^ cells: medium vs. DSP216a p≤0.0001^****^, medium vs. DSP216i *p* = 0.9135 ns, DSP216a vs. DSP216i *p* = 0.0003^***^; HT1080^wt^: DSP216a vs. DSP216i *p* = 0.0635 ns; Jurkat‐Lucia^TM^ NFAT‐CD16 only DSP216a vs. DSP216i *p* = 0.1118 ns). (D) Percentage of BV421 Annexin V^+^ 721.221^EV^ cells (white symbols) or 721.221^HLA‐G^ cells (midnight blue symbols) in coculture with NK cells at different effector: target ratios. Each line represents an individual donor (biological replicate). Parametric paired t‐test was used to test if the difference between treated and untreated samples was significant (16:1 *p* = 0.0012^**^, 8:1 *p* = 0.0025^**^, 4:1 *p* = 0.0032^**^, 2:1 *p* = 0.0014^**^). (E) Percentage of BV421 Annexin V^+^ 721.221^HLA‐G^ cells in coculture with NK cells at different effector : target ratios without (light blue symbols) or with 10 µg/mL DSP216i (dark blue symbols). Samples were analyzed is in (D)(16:1 *p* = 0.2550 ns, 8:1 *p* = 0.0774 ns, 4:1 *p* = 0.0182^*^, 2:1 *p* = 0.0243^*^). (F) Percentage of BV421 Annexin V^+^ 721.221^EV^ cells in coculture with NK cells at different effector : target ratios without (light blue symbols) or with 10 µg/mL DSP216i (dark blue symbols). Samples were analyzed as in (D) (16:1 *p* = 0.1790 ns, 8:1 *p* = 0.3385 ns, 4:1 0.0823 ns, 2:1 *p* = 0.0778 ns). (G) Percentage of BV421 Annexin V^+^ 721.221^HLA‐G^ cells in coculture with NK cells at different effector : target ratios without (white symbols) or with 10 µg/mL DSP216a (gray symbols). Samples were analyzed as in (D)(16:1 *p* = 0.0385^*^, 8:1 *p* = 0.0171^*^, 4:1 *p* = 0.0423^*^, 2:1 *p* = 0.0575 ns). (H) Percentage of BV421 Annexin V^+^ 721.221^EV^ cells in coculture with NK cells at different effector : target ratios without (white symbols) or with 10 µg/mL DSP216a (gray symbols). Samples were analyzed is in (D)(16:1 *p* = 0.2104 ns, 8:1 *p* = 0.5211 ns, 4:1 *p* = 0.3044 ns, 2:1 *p* = 0.5008 ns). (I) Mean increase in % BV421 Annexin V^+^ 721.221^HLA‐G^ cells with DSP216i (blue bar) or DSP216a (gray bar). An unpaired parametric t‐test was used to test if the difference in the increase of NK cytotoxicity was significant. Sample size DSP216i *n* = 6 and sample size DSP216a *n* = 4 (16:1 *p* = 0.0207^*^, 8:1 *p* = 0.0881 ns, 4:1 0.2505 ns, 2:1 *p* = 0.1954 ns). ^*^ *p* < 0.05; ^**^ *p* < 0.01; ^***^ *p* < 0.001; ^****^ *p* < 0.0001.

To characterize FcR triggering mediated by DSP216, Jurkat‐LuciaNFAT‐CD16 cells, in which a luminescent signal depends on CD16‐mediated FcR engagement (Figure 5A), were cocultured with HT1080^HLA‐G^ cells and treated with DSP216i or DSP216a. DSP216a treatment induced a significant and dose‐dependent increase in luminescence (Figure 5B), whereas treatment with DSP216i did not significantly trigger luminescence compared to control treatment, even at the highest doses (Figure 5B). DSP216a induced significant luminescence in coculture only with HT1080^HLA‐G^ cells, but not HT1080^wt^ cells or Jurkat monocultures. Further, and in line with the anticipated FcR‐dependent mode of action, DSP216i did not significantly induce luminescence under any of the conditions (Figure 5C). Thus, DSP216a, but not DSP216i, was able to activate CD16 upon coordinate binding to HLA‐G and CD47.

HLA‐G serves as an inhibitory checkpoint for NK cells, with binding of HLA‐G to LILRB1 on NK cells previously shown to reduce NK cytotoxicity against target cells. [30] As described above, DSP216 is designed, by virtue of its LILRB2 domain, to also inhibit HLA‐G/LILRB1 interaction. Correspondingly, HLA‐G expression significantly reduced NK‐mediated killing of target cells by primary NK cells in coculture assays (Figure 5D). Both DSP216i and DSP216a each reversed this HLA‐G checkpoint activity and significantly increased NK cell killing of 721.221^HLA‐G^ cells at two of four and three of four effector:target (E:T) ratios, respectively (Figures 5E,G, for gating strategy see Figure S13 and for representative flow cytometry plots see Figure S14). Importantly, neither DSP216i nor DSP216a had a significant effect on NK cytotoxicity toward 721.221^EV^ cells under the current experimental conditions (Figures 5F,H). The increase in NK‐mediated killing of target cells was significantly higher with DSP216a than with DSP216i at one of four E:T ratios, with a trend toward a higher increase at the remaining E:T ratios (Figure 5I). Thus, our data demonstrate that each of DSP216i and DSP216a can block HLA‐G‐mediated inhibitory signaling toward NK cells expressing LILRB1 as a receptor, and DSP216a additionally induces NK‐mediated killing by activating CD16.

## 3 Discussion

In this study, we describe the design of a dual signaling protein, DSP216, comprising the ECDs of human SIRPα and LILRB2, with a single amino acid substitution (V78R) in the LILRB2 ECD to increase its affinity for HLA‐G, fused through heterodimerizing complementary knob‐in‐hole IgG1 Fc backbones. Our data documents the AND‐gate binding property of DSP216, as it selectively binds to cells co‐expressing HLA‐G and CD47, but only marginally to cells expressing CD47 only, such as RBCs and PBMCs. Consistent with this binding profile, DSP216 outcompeted soluble SIRPα binding to, and increased phagocytosis of, HLA‐G and CD47 double‐expressing cancer cells, but not cells expressing CD47 only. Further, DSP216 treatment enhanced NK‐mediated cytotoxicity of cancer cells co‐expressing HLA‐G and CD47 and prevented HLA‐G‐mediated (M2) polarization of macrophages in cocultures with HLA‐G and CD47 double expressors. In addition, DSP216 with an active Fc domain activated the CD16 Fc receptor in a reporter assay and further increased NK cell‐mediated killing of double expressors, but not single expressors. Thus, DSP216a combined checkpoint blockade with FcR triggering capacity.

Our data establish that DSP216 is an ICI that effectively blocks both the CD47/SIRPα and the HLA‐G/LILRB1/2 axis. DSP216 has clear advantages compared to other agents targeting the LILRB1/2/HLA‐G axis in clinical trials—HLA‐G Ab TTX‐080, LILRB1 Ab BND‐22, and LILRB2 Ab JTX‐8064. In contrast to an HLA‐G Ab, DSP216, by invoking an HLA‐G counter‐receptor, targets multiple HLA‐G isoforms (see Introduction). [10] Whereas LILRB1‐ and LILRB2‐directed Abs can block HLA‐G signaling from multiple isoforms, they also block inhibitory signaling of normal cells expressing classical HLA‐A molecules. [31] Further, DSP216 blocks both LILRB2/HLA‐G and LILRB1/HLA‐G axes, whereas LILRB1/2 Ab blocks merely one of the ligands, respectively. Given that HLA‐G is a well‐established tumor‐selective target antigen, this mode of action confers safety. In contrast, LILRB1/2 Abs bind LILRB1/2 expressed on immune cells throughout the body, [31] with LILRB1 even overexpressed in the peripheral blood of cancer patients. [32] The additional inherent advantage of DSP216 is that it acts as a dual checkpoint inhibitor that also alleviates the CD47/SIRPα axis, promoting innate immune response in a multifold manner.

We further demonstrated that DSP216 promotes phagocytosis of and NK cytotoxicity against CD47 and HLA‐G double‐expressing cell lines. The LILRB1 Ab BND‐22 also promotes phagocytosis of and NK cytotoxicity toward HLA‐G‐expressing cell lines and activates LILRB1‐transduced Jurkat cells (T cell line) in cocultures with HLA‐G‐expressing cells. [32] However, unlike DSP216, it promotes phagocytosis and NK cytotoxicity more potently in control target cells lacking HLA‐G expression. [32] This HLA‐G‐independence of BND‐22 Ab suggests that it might rely on blocking the interaction between LILRB1 and classical HLA molecules, which in turn could increase the risk of adverse events. This is not the case for DSP216.

The same issue surrounds the LILRB2 Ab JTX‐8064, which blocks binding of HLA‐G tetramers to not only human monocyte‐derived macrophages (HMDMs), but also to classical HLA molecule HLA‐A2. [31] Of note, JTX‐8064 Ab, like DSP216, promotes production of the pro‐inflammatory cytokine (TNFα) and reduces the production of the anti‐inflammatory cytokine IL‐10 by HMDMs. Gene expression analysis of these macrophages revealed that several M1‐associated molecules were upregulated and M2‐associated ones were downregulated. Yet, no data was provided as to whether JTX‐8064 Ab induces direct cytotoxicity towards cancer cells, as does DSP216. Also of note for the HLA‐G‐directed Ab TTX‐080, no preclinical data are available, and it is unclear if Tizona Therapeutics overcame the hurdle that an HLA‐G Ab, in contrast to DSP216, generally targets only one HLA‐G isoform. [10]

In addition to the LILRB1/2/HLA‐G axis, DSP216 also blocks the CD47/SIRPα axis. We hypothesize that parallel blocking of these two checkpoints might have a synergistic effect. Promoting phagocytosis of cancer cells co‐expressing HLA‐G and CD47 could potentially lead to subsequent priming of cancer antigen‐specific T cells. Coupling this to the abrogation of HLA‐G‐mediated inhibitory effects on T cells could thus synergistically support the restoration of the cancer immunity cycle and could ultimately contribute to the elimination of CD47 (single) positive cancer cells if present in a tumor. The extent to which DSP216 can indeed reverse HLA‐G‐mediated inhibition of adaptive immune cells, specifically T cells, requires further evaluation.

As described in the introduction, targeting of CD47 with monospecific molecules, such as magrolimab, has faced challenges. [11] During Phase I and II trials, side effects, such as anemia stemming from binding of the CD47 Ab to CD47 expressed on normal blood cells, seemed manageable. However, in early 2024, all magrolimab trials were discontinued due to a higher risk of death in treatment groups as compared to control groups. [20] DSP216 is configured to minimize binding to cells expressing CD47 only, with two key design features. First, DSP216 binds CD47 via a counter‐receptor (SIRPα domain), as opposed to an CD47 Ab, which was previously shown not to induce anemia in patients, as interaction with RBCs is minimal. Indeed, the SIRPα‐Fc fusion protein TTI‐621 and SIRPα‐Fc‐CD40L fusion protein SL‐172154, as well as the SIRPα fusion protein DSP107, do not induce hemolytic anemia in patients. [33, 34, 35] There are several hypotheses that could explain this observation: SIRPα might only bind sufficiently to CD47 clusters, which are not present on RBCs or, alternatively, the conformational state of CD47 might hinder SIRPα binding. [36, 37] Second, as we have demonstrated in the present study, DSP216 binds only with sufficient strength to HLA‐G and CD47 double‐positive cells through the so‐called ‘AND‐gate’. The high(er) specificity towards cancer cells due to this AND‐gate binding property might not only reduce side effects, but also reduce CD47 antigen sink on normal tissues and, thereby, enable a reduction in treatment dose. Indeed, it was shown that a ten times lower dose of the SIRPα fusion proteins DSP107 and SL‐172154 (which do not bind CD47 on RBCs) was needed than for magrolimab (which does bind CD47 on RBCs), to reach 100% receptor occupancy on circulating WBC (3 mg/kg [34, 35] vs. 30 mg/kg [38]).

Various CD47 blocking agents with active Fc domains, mostly IgG1, as well as inactive Fc domains, mostly IgG4, are under clinical investigation. Whereas CD47‐targeting agents not engaging Fc receptors were initially reported as well‐tolerated, but not efficient as monotherapy, CD47‐targeting agents with an Fc domain engaging Fc receptors showed some therapeutic activity that was also associated with dose‐limiting on‐target/off‐tumor effects. The fact that CD47‐blocking agents without Fc receptors engaging Fc domains do synergize with antitumor Ab, for instance DSP107 with RTX [1], suggests that it is desirable to combine CD47 blocking with Fc‐mediated cytotoxicity. As the side effects of agents with active Fc arise from on‐target/off‐tumor binding, the unique selective binding of DSP216a might position it as an advantageous CD47‐targeting agent with a good toxicity profile.

Animal models are currently considered the gold standard for efficacy and safety of drugs. However, the EMA as well as the FDA encourage the use of new approach methodologies (NAMs) to replace animal studies in clinical trial applications (CTA) or investigational new drug applications (INDs), respectively [39, 40], addressing the fact that 80–90% of new drugs fail in clinical trials due to lack of clinical efficacy or toxicity. [41] HLA‐G is not preserved in mice and non‐human primates, making these species non‐relevant animals for toxicity studies. In these cases, a package including *in* *vitro* studies with human‐derived cells and PK data from non‐terminal cynomolgus monkey PK studies should be submitted to get approval for a phase I study from the authorities, as exemplified for the biscpeific T cell engager tebentafusp. [42] The data reported here supports the preparation of such a package for DSP216 and will contribute to enabling clinical trials with DSP216 in the near future.

## 4 Conclusion

The fusion protein DSP216 binds exclusively to cells co‐expressing HLA‐G and CD47, but only marginally to RBCs and PBMCs expressing CD47 only. Functional assays confirmed that not only DSP216's binding profile, but also its anti‐cancer and immunostimulatory activities follow an AND‐gate mode, requiring the presence of both molecular targets on cancer cells. Thus, DSP216 represents a novel dual checkpoint inhibitor that combines CD47 and HLA‐G blockade to promote anti‐cancer immunity through activation of macrophages and NK cells. This effect can be further enhanced by immune cell activation mediated through its Fc domain.

## 5 Experimental Section/Methods

### 5.1 LILRB2 Sequence and Optimization

Prior to the fusion protein design steps, crystal structures of proteins were prepared (fixing charges and missing side chains) and energy minimization was performed for the complex structure. CHARMM‐based force‐field energy calculation was used to iteratively predict the contribution of mutations in LILRB2 sequence to affinity for HLA‐G, while preserving protein stability. The process involved the following steps: First, structural analysis was performed to identify potential interaction points between LILRB2 and HLA‐G with the high‐resolution complex structure (PDB ID: 2DYP). Second, sequences between HLA homologues HLA‐A, HLA‐B, HLA‐C and HLA‐G were compared to identify differences in the interacting sites, which are specific for the LILRB2‐HLA‐G interaction. Third, a subset of residues defining the interface between LILRB2 and HLA‐G were extracted for mutational analysis. Fourth, each possible mutation in the subset of residues was sampled. This process involved introduction of specific substitutions at each position separately, optimization of the structure to best accommodate the mutation and calculation of the energy difference (ΔG vs. wt.) either on the entire protein stability or on binding energy. Fifth, the results of the binding/stability energy calculations were compiled into a ranked list and point mutations on the LILRB2 sequences predicted to be beneficial for both binding and stability were selected. Sixth, these selected mutations were used in a second iteration of mutational analysis, this time introducing two mutations each time and repeating the process of energy evaluation on both binding to HLA‐G and stability. The 10 highest ranked substitutions or combinations of substitutions were produced at small‐scale and used for binding assays (Table S1/S2).

### 5.2 Reagents

#### 5.2.1 Proteins and Monoclonal Antibodies (mAbs)

CD47‐Fc (IgG1) was produced at GenScript (sequence provided in Document S2), HLA‐G‐his (cat#ab225660, abcam), Mouse Anti‐Human IgG4 Fc Ab‐HRP (cat# 9200–05, Southern Biotech), CD47 mAb (sequence from patent [43], huIgG4) and HLA G mAb (sequence from patent [44], hIgG4) were produced at KAHR lab; His‐tagged SIRPα (cat#SIA‐H5225‐100ug, ACRO Biosystems); His‐tagged LILRB2 (cat#8429‐T4, lot#DECE022341) and IL‐10 (R&D systems); GM‐CSF (cat#11343127), M‐CSF (cat#11343117), IFN‐γ (cat#11343536, Immunotools); LPS (cat#L5293‐2ML, Sigma‐Aldrich).

#### 5.2.2 Flow Cytometry Reagents

AF647‐IgG1 hinge antibody (cat#9052‐31, clone 4E3, lot#H0321‐PK42G), AF647‐IgG4Fc (cat# 9200–31, clone HP6025, lot#L2621‐ND72C, Southern Biotech); AF647‐Penta‐His antibody (cat#35370, lot#175028589, Qiagen); Fc blocking solution Human TruStain FcX (cat#422302, lot#B328706), BV421‐CD45 Ab (cat#304032, clone HI30, lot#B336863), BV605‐CD45 Ab (cat#304042, clone HI30, lot#B368891), APC‐CD16 Ab (cat#360706, clone B73.1, lot#B303131), BV785‐CD11b Ab (cat#301346, clone ICRF44, lot#B402129), APC‐CD163 Ab (cat#333610, clone GHI/61, lot#B380802), APC‐CD14 Ab (cat#367118, clone 63D3, lot#B275678), APC‐CD47 Ab (cat#323124, clone CC2C6, lot#B313461), APC‐CD11b Ab (cat#101212, clone M1/70, lot#B384396), APC‐IgG1 isotype ctrl (cat#400120, clone MOPC‐21, lot#B299375), Pacific Blue Annexin V Apoptosis Detection Kit with 7‐AAD (cat#640926, Biolegend); BD Horizon Fixable Viability Stain 450 (cat#562247, BD); CellTrace Violet Cell Proliferation Kit (cat# C34557), CellTrace CFSE Cell Proliferation Kit (cat#C34554, Thermo Fisher); FACS buffer (in house, PBS with 0.5% BSA, 2 mM EDTA, 0.05% sodium azide). Unless described otherwise, staining was performed in 96‐well plates in 50 µL FACS buffer.

#### 5.2.3 Primary Cells

Buffy coats were obtained from Sanquin, NL (agreement number NVT0465.01) or Hadassah blood bank, IL (Helsinki No. 0155‐17‐HMO); Ficoll‐Paque (GE) or Lymphoprep (Serumwerk Bernburg AG); RBC lysing buffer (Roche); RosetteSep Human NK Cell Enrichment Cocktail (STEMCELL Technologies).

#### 5.2.4 Other

1‐Step Ultra TMB‐ELISA Substrate Solution (cat#T0440‐100 mL Sigma), TMB stop solution (450 nm, cat#0412‐01, Southern Biotech), BSA (cat#03‐010‐1B, BI)Alexa Fluor 647 Conjugation Kit (Fast)—Lightning‐Link (Abcam); BDTM Cytometric Bead Array (CBA) Human Th1/Th2 Cytokine Kit (BD biosciences); IIQUANTI‐Luc assay reagent (InvivoGen)

### 5.3 Cell Lines and Culture Conditions

Expi293F cells were purchased from Gibco and cultured in Expi293 medium (A14527, Gibco). 721.221 cells were obtained from the Lotem lab (RRID: CVCL_6263). [44] HT1080 cells (cat#CCL‐121, RRID: CVCL_0317) and JEG‐3 cells (cat#HTB‐36, RRID: CVCL_0363) were purchased from ATCC. HLA‐G overexpressing or empty vector (EV) cell lines were produced by lentiviral transduction with pRRL‐SFFV‐HLA‐G‐iGFP or pRRL‐SFFV‐iGFP plasmid. Lentiviral particles were produced and used to transduce cells as described before. [1] 721.221^EV^ and 721.221^HLA‐G^ cells were cultured in RPMI medium (Gibco) supplemented with 10% FCS. HT1080^wt^ and HT1080^HLA‐G^ cells were cultured in EMEM medium (Gibco) and DMEM medium (Gibco), respectively, supplemented with 10% FCS, 1% glutamax and 1% PenStep. Clones of HT1080^HLA‐G^ cells were generated by limiting dilution. JEG‐3 cells were cultured in EMEM medium supplemented with 10% FCS. Jurkat‐Lucia NFAT‐CD16 cells were part of JKTL‐NFAT‐CD16 kit purchased from InvivoGen and were grown in RPMI medium supplemented with 10% FCS, 1% glutamax, 1% PenStrep, 10 µg/mL Blasticidin, 100 µg/mL Zeocin, 100 µg/mL Normocin. All cell lines were regularly tested to be mycoplasma‐free.

### 5.4 Isolation and Culture of Immune Cells

PBMCs were isolated from buffy coat by density gradient centrifugation. To this end, a buffy coat was diluted 1:1 with PBS and layered carefully on top of lymphoprep/Ficoll‐Paque at ratio 2:1 (diluted blood: lymphoprep/Ficoll‐Paque) in a 50 mL tube. After 20 min centrifugation (800 × g, no break), PBMCs in the layer between lymphoprep and plasma were aspirated, washed with PBS (5 min, 800 × g), remaining erythrocytes were lysed with RBC lysis buffer and PBMCs were washed again with PBS (10 min, 350 × g). PBMCs were seeded in RPMI medium (Gibco) in T75 cell culture flasks (ThemoScientific Nunc) for 2 h whereupon monocytes attached, and non‐adherent cells were removed. Monocytes were differentiated to immature macrophages (iM) by 6‐day culture in RPMI medium supplemented with 10% FCS and M‐CSF (50 ng/mL) with medium being renewed every two days. In the final polarization step, iM were cultured for 24 h in RPMI medium supplemented with 10% FCS, LPS (100 ng/mL) and INFγ (50 ng/mL) to generate mature M1‐like macrophages (M1) and with RPMI medium supplemented with 10% FCS and IL‐10 (100 ng/mL) to generate mature M2‐like macrophages (M2). NK cells were isolated from full blood by using an immunodensity negative selection cocktail according to manufacturer's instructions (unwanted cells are cross‐linked to red blood cells during density gradient separation) and NK cells were cultured for up to 48 h in ex‐vivo medium (Lonza).

### 5.5 DSP216 Production and Characterization

For comparative functional analysis and production evaluation, several recombinant proteins comprising a wild type (wt) LILRB2 domain with IgG4 Fc (LILRB2‐WT‐linker‐IgG4 T366S L368A Y407V hole Fc [45], Doc S1 Sequence 1) or IgG1 Fc with LALA mutations (LILRB2‐WT‐linker‐IgG1 T366S L368A Y407V hole Fc [45], Doc S1 Sequence 2) or a mutated LILRB2 domain (LILRB2‐V78R‐linker‐IgG1 Y249C T366S L368A Y407V hole Fc [45], Doc S1 Sequence 3) were produced together with a SIRPα domain with an IgG4 Fc (SIRPα‐linker‐IgG4 S354C T366W knob Fc, DocS1 Sequence 4) or IgG1 Fc (SIRPα‐linker‐IgG1 S354C T366W knob Fc, Doc S1 Sequence 5). Alternatively the IgG1 domains were produced with L234A L235A (LALA) mutations (Doc S1 Sequences 6–8). [46] Production was performed in Expi293F cells transiently transfected by pcDNA3.4 expression vectors (ratio 1:1, cat#A14697, ThermoFisher) cloned with the coding sequence for the desired Fc fusion proteins. The sequences were cloned into the vector using restriction enzymes EcoRI and HindIII or XbaI and EcoRV, with addition of a Kozak sequence and an artificial signal peptide. The heteromonomers were co‐expressed and collected from the supernatant of cell culture, purified by using protein A (PA) chromatography (Poros MabCapture A resin or TOSOH TOYOPEARL AF‐rProtein A HC‐650F or Cytiva MabSelect SuRe) and, for purification of large batches after variant screening, size exclusion chromatography (SEC) (Cytiva Superdex 200 resin). For some experiments DSP216 was labelled with the fluorochrome AF647 using the Alexa Fluor 647 Conjugation Kit (Fast)—Lightning‐Link kit according to manufacturer's instructions.

### 5.6 ELISA

Binding of DSP216 to recombinant human HLA‐G and CD47 was assessed by ELISA. 96‐well plates were coated overnight with 70 µL/well CD47‐Fc (1 µg/mL) or HLA‐G‐his (5 µg/mL, abcam) in PBS at 4°C and subsequently blocked for 1 h with 200 µL 1% BSA in washing buffer (WB, PBST 0.05%). 50 µL DSP216 (IgG4, 0–50 µg/mL) was added to the wells and incubated for 2 h. Then plate‐bound DSP216 was detected by incubation with Mouse Anti‐Human IgG4 Fc Ab‐HRP (100 µL/well, 1:20000). Each incubation was followed by three washes with 300 µL/well WB. For detection, 100 µL/well TMB substrate (Sigma) was added and reaction was stopped with 100 µL TMB stop solution (SouthernBiotech). Absorbance at 450 and 620 nm was measured by plate reader (Multiskan FC 96 Plate Microplate Photometer, Thermo Fisher Scientific) and final absorption was calculated as follows:

 absorption = (absorption sample 450 nm‐absorption sample 620 nm)‐(absorption blank450 nm‐absorption blank 620 nm)

All samples were analyzed in triplicates.

### 5.7 Binding of DSP216

Binding of DSP216 to cancer cells was assessed by seeding cells in a 96‐well plate (5 × 10^4^ cells/well) with 50 µL cell culture medium containing different concentrations of DSP216 (0‐25 µg/mL) for 30 min at 4°C, followed by one wash with 150 µL FACS buffer (450 × g, 5 min). Cell‐bound DSP216 was detected by incubation with human AF647‐IgG1 hinge antibody (250 ng/well) for 30 min at 4°C. After two washes with 150 µL FACS buffer (450 × g, 5 min), binding was analyzed by flow cytometry (NovoCyte Quanteon Flow Cytometer Systems 4 Lasers, Agilent). To test specificity of DSP216 binding to HLA‐G and CD47, target cells were pre‐incubated with HLA‐G (IgG4, 500 ng/well) and/or CD47 (IgG4, 500 ng/well) blocking antibodies in 50 µL culture medium for 1 h at 37°C, before addition of 50 µL culture medium containing DSP216. Further, selective binding of DSP216 was assessed by mixing HT1080^HLA‐G^ cells (2.5 × 10^4^ GFP^+^/well) with PBMCs (2.5 × 10^4^/well) and 100 µL of 1:500 diluted whole blood (containing approximately 1 × 10^6^ RBCs), whereupon cells were pre‐incubated with Fc blocking solution (2.5 µL diluted stock solution/well) for 15 min at room temperature (RT) before addition of AF647‐labelled DSP216 (12.5 µg/mL) and incubation for 30 min at RT. After washing the cells with 150 µL FACS buffer (450 × g, 5 min), cells were stained with BV421‐CD45 Ab (12.5 ng/well) for 30 min at 4°C, washed again with 150 µL FACS buffer (450 × g, 5 min), and analyzed by flow cytometry (CytoFLEX system B5‐R3‐V5, BC‐53000, Beckman Coulter). To test if DSP216 inhibited binding of SIRPα to CD47, 721.221^EV^/721.221^HLA‐G^ cells were seeded in 96‐well plates (5 × 10^4^ cells/well) in 100 µL cell culture medium containing different concentrations of DSP216 (0‐2080 nmol/L) and His‐tagged SIRPα (260 nmol/L) for 30 min at 4°C. After a washing with 150 µL FACS buffer (450 × g, 5 min), cells were incubated 30 min at 4°C with APC Penta‐His antibody (125 ng/well) to detect cell‐bound SIRPα. Following a washing step with 150 µL FACS buffer (450 × g, 5 min), the cells were analyzed by flow cytometry (NovoCyte Quanteon Flow Cytometer Systems 4 Lasers, Agilent).

### 5.8 PBMC Viability Assay

Cytotoxicity of DSP216 was tested using PBMCs from buffy coats of six healthy donors. PBMCs were seeded in 96‐well plates (1 × 10^5^ cells/well) and incubated with serial dilutions of DSP216 (0‐25 µg/mL) in culture medium for 24 h or 48 h at 37°C, 5% CO2. Following incubation, cells were washed with 150 µL FACS buffer (450 × g, 5 min), stained with viability dye for 15 min at RT (0.05 µL per well), washed with 150 µL FACS buffer (450 × g, 5 min), pre‐incubated for 15 min at RT with Fc blocking solution (2.5 µL diluted stock solution/well) and stained with BV605‐hCD45 Ab (50 ng/well) for 30 min at 4°C. Following incubation, cells were washed with 150 µL FACS buffer (450 × g, 5 min) and the percentage of live CD45^+^ cells was analyzed by flow cytometry (CytoFLEX system B5‐R3‐V5, BC‐53000, Backman Coulter).

### 5.9 Effect of DSP216 on Macrophage Polarization

Effect of DSP216 on macrophages cocultured with HLA‐G and CD47 expressing cells was assessed by seeding iM2 macrophages in 6‐well plates (2.5 × 10^5^/well) overnight (37°C, 5% CO_2_). On the next day, GFP^+^ HT1080^HLA‐G^ cells were pre‐incubated with DSP216 (10 µg/mL) or HLA‐G Ab (1.5 µg/mL) for 1 h at 37°C after which HT1080^HLA‐G^ cells were seeded on top of the macrophages in the 6‐well plates (1.25 × 10^5^ cells/well). Following 24 h incubation, supernatants were collected and analyzed for secreted cytokines using a Cytometric Bead Array kit according to manufacturer's instructions.

The remaining cells were detached and seeded in 96‐well plates (5 × 10^4^ cells/well), washed with 150 µL PBS (450 × g, 5 min) and stained with viability dye for 15 min at RT (0.05 µL per well). Then cells were washed with 150 µL FACS buffer (450 × g, 5 min), incubated with Fc blocking solution for 15 min at RT (1 µL diluted stock solution/well) and stained with BV785‐CD11b Ab (50 ng/well) and APC‐CD163 Ab (50 ng/well) or APC‐CD14 Ab (100 ng/well). Following two washing steps with 150 µL FACS buffer (450 × g, 5 min), cells were analyzed by flow cytometry (CytoFLEX system B5‐R3‐V5, BC‐53000, Backman Coulter).

### 5.10 Macrophage‐mediated Phagocytosis

To assess the phagocytic uptake of cancer cells (721.221^EV^/721.221^HLA‐G^ or JEG‐3 cells) by M2c macrophages, CTV stained cancer cells (staining according to manufacturer's protocol) were seeded in FACS tubes (15 × 10^4^/tube) and pre‐incubated with 2.5/5/10 µg/mL DSP216 or 6 µg/mL mCD47 Ab for 20 min at RT before M2c were added on top (5 × 10^4^ M2c macrophages/tube, resulting in Effector: Target (E:T) ratio 1:3 and a total volume of 200 µL). After 3 h incubation at 37°C 5% CO_2_, the cell mixture was incubated with CD11b‐APC Ab (150 ng/tube) and the fraction of CD11b^+^ CTV^+^ macrophages was determined by flow cytometry (NovoCyte Quanteon Flow Cytometer Systems 4 Lasers, Agilent).

### 5.11 ADCC Reporter Assay

To assess if DSP216 with active and inactive Fc could activate the CD16 reporter cell line, target cells (10 × 10^4^/well), either HT1080^wt^ or HT1080^HLA‐G^ cells, were seeded in 96‐well plates in 50 µL cell culture medium containing different concentrations of DSP216 (0‐6.7 µg/mL) and incubated for 1 h at 37°C. Subsequently, JurkatCD16‐NFAT effector cells (20 × 10^4^/well) were added on top of the target cells in 50 µL cell culture medium and incubated for 5 h at 37°C. In control conditions, only effector cells were incubated with DSP216. Luciferase assay was performed according to JKTL‐NFAT‐CD16 kit protocol. Briefly, the supernatants (20 µL/well) were transferred into 96‐well white Opti plate (Greiner Bio‐one), then QUANTI‐Luc assay reagent (50 µL/well) was added, and signal was measured in luminometer plate reader (Thermo, Flouroskan FL).

### 5.12 NK Cytotoxicity Assay

NK‐mediated cytotoxicity was tested by pre‐incubating CFSE stained 721.221^EV^/721.221^HLA‐G^ cells (staining according to manufacturer's protocol) in FACS tubes (2 × 10^4^/tube) with culture medium containing DSP216 (0 µg/mL or 10 µg/mL) for 20 min at RT before NK cells (3.2/1.2/0.8/0.4 × 10^5^/tube) were added (resulting in E:T ratios 16:1/8:1/4:1/ 2:1 and total volume 200 µL). After 3 h incubation at 37°C 5% CO_2_ and one washing step with 3 mL FACS buffer (450 × g, 5 min) the cell mixture was incubated with BV421 Annexin V solution (100 µL, 1 µL stock solution in in 100 µL Annexin V binding buffer) for 15 min at RT before addition of Annexin V binding buffer (400 µL) to quench the staining. 200 µL/tube were analyzed by flow cytometry (NovoCyte Quanteon Flow Cytometer Systems 4 Lasers, Agilent) and the fraction of CFSE+ 721.221^EV^/721.221^HLA‐G^ cells in early apoptosis was determined.

### 5.13 Statistical Analysis

Where indicated, binding data were normalized with 100% defined as indicated in the figure legend. No statistical tests were performed on normalized data. Sample size for each statistical analysis is given in the figure legend. Statistical analysis was performed by two‐sided parametric paired or unpaired t‐test as indicated in the figure legend, using GraphPad Prism (GraphPad Software 10.2.3, La Jolla, CA). Where indicated, ^*^ *p* < 0.05; ^**^ *p* < 0.01; ^***^ *p* < 0.001; ^****^ *p* < 0.0001. All the data were presented as mean + standard deviation.

### 5.14 Ethics Statement

All procedures were performed in accordance with International Ethical and Professional Guidelines (the Declaration of Helsinki and the International Conference on Harmonization Guidelines for Good Clinical Practice). Human blood samples received from Sanquin (NL) and used under agreement number NVT0465.01 and from Hadassah blood bank, IL under Helsinki No. 0155‐17‐HMO

## Funding

This research has received funding from the European Union´s Horizon 2020 research and innovation program under the Marie Sklodowska‐Curie grant agreement No 813871 and from KWF grant agreement No15539.

## Conflicts of Interest

E.B.s research is financially supported by Kahr Medical; L.J., L.T, M.A, A.T., R.K., I.P., Y.P. and A.C. are/were employees of Kahr Medical; E.B., L.T., A.T., R.K., I.P, Y.P. and A.C. are shareholders of Kahr Medical.

## Supporting information




**Supporting File**: advs74318‐sup‐0001‐SuppMat.docx.

## Data Availability

All data generated or analyzed during this study are included in this published article [and its supplementary information files].

## References

[1] E. Cendrowicz , L. Jacob , S. Greenwald , et al., “DSP107 combines inhibition of CD47/SIRPα axis With activation of 4‐1BB to trigger anticancer immunity,” Journal of Experimental & Clinical Cancer Research 41, no. 1 (2022): 97, 10.1186/s13046-022-02256-x. 35287686 PMC8919572

[2] J. J. Luke , A. Saeed , B. Bashir , et al., “Phase 1 Dose Escalation Study of DSP107, a first‐in‐class CD47 and 4‐1BB Targeting Multifunctional immune‐recruitment Protein, in patients with advanced solid tumors,” 2022: 2647–2647, 10.1200/JCO.2022.40.16_suppl.2647.

[3] S. Wang , J. Wang , Y. Xia , et al., “Harnessing the potential of HLA‐G in cancer therapy: Advances, challenges, and prospects,” Journal of Translational Medicine 22, no. 1 (2024), 10.1186/s12967-024-04938-w. PMC10838004 38310272

[4] V. Cirulli , J. Zalatan , M. McMaster , et al., “The class I HLA Repertoire of Pancreatic Islets Comprises the Nonclassical Class Ib Antigen HLA‐G,” Diabetes 55, no. 5 (2006): 1214–1222, 10.2337/db05-0731. 16644675

[5] M. Le Discorde , P. Moreau , P. Sabatier , J.‐M. Legeais , and E. D. Carosella , “Expression of HLA‐G in Human Cornea, an Immune‐Privileged Tissue,” Human Immunology 64, no. 11 (2003): 1039–1044, 10.1016/j.humimm.2003.08.346. 14602233

[6] V. Mallet , A. Blaschitz , L. Crisa , et al., “HLA‐G in the Human Thymus: A Subpopulation of Medullary Epithelial but not CD83+ Dendritic Cells Expresses HLA‐G as a Membrane‐bound and Soluble Protein,” International Immunology 11, no. 6 (1999): 889–898, 10.1093/intimm/11.6.889. 10360962

[7] P. Paul , N. Rouas‐Freiss , I. Khalil‐Daher , et al., “HLA‐G Expression in Melanoma: A Way for Tumor Cells to Escape from Immunosurveillance,” Proceedings of the National Academy of Sciences 95, no. 8 (1998): 4510–4515, 10.1073/pnas.95.8.4510. PMC22520 9539768

[8] Tizona Therapeutics , Tizona Initiates Clinical Development of TTX‐080 in Advanced Cancers: ‐IND Cleared by US FDA With Study Enrollment to Begin in Q3 2020 ‐First Anti‐HLA‐G Antibody Into the Clinic. (Tizona Therapeutics 2020), https://www.tizonatx.com/news/press‐releases/062420/.

[9] Tizona Therapeutics , Tizona Initiates Phase 1b Expansion Study of TTX‐080 in Advanced Refractory or Resistant Malignancies: Expansion study will evaluate TTX‐080, an anti‐HLA‐G Antibody, as monotherapy and in combination With either pembrolizumab or cetuximab in solid tumors (Tizona Therapeutics 2021), https://www.tizonatx.com/news/press‐releases/101421/.

[10] M. Shiroishi , K. Kuroki , L. Rasubala , et al., “Structural Basis for Recognition of the Nonclassical MHC Molecule HLA‐G by the Leukocyte Ig‐like Receptor B2 (LILRB2/LIR2/ILT4/CD85d),” Proceedings of the National Academy of Sciences 103, no. 44 (2006): 16412–16417, 10.1073/pnas.0605228103. PMC1637596 17056715

[11] R. Bouwstra , T. van Meerten , and E. Bremer , “CD47‐SIRPα Blocking‐based Immunotherapy: Current and Prospective Therapeutic Strategies,” Clinical and Translational Medicine 12, no. 8 (2022), 10.1002/ctm2.943. PMC9339239 35908284

[12] M. E. W. Logtenberg , F. A. Scheeren , and T. N. Schumacher , “The CD47‐SIRPα Immune Checkpoint,” Immunity 52, no. 5 (2020): 742–752, 10.1016/j.immuni.2020.04.011. 32433947 PMC7340539

[13] J. Huang , F. Liu , C. Li , et al., “Role of CD47 in Tumor Immunity: A Potential Target for Combination Therapy,” Scientific Reports 12, no. 1 (2022), 10.1038/s41598-022-13764-3. PMC9192775 35697717

[14] M. Hernandez‐Gamarra , A. Salgado‐Roo , E. Dominguez , et al., “CARTAR: A Comprehensive Web Tool for Identifying Potential Targets in Chimeric Antigen Receptor Therapies using TCGA and GTEx Data,” Briefings in Bioinformatics 25, no. 4 (2024), 10.1093/bib/bbae326. PMC11229032 38975894

[15] S. E. Kauder , T. C. Kuo , O. Harrabi , et al., “ALX148 Blocks CD47 and Enhances Innate and Adaptive Antitumor Immunity with a Favorable Safety Profile,” PLoS ONE 13, no. 8 (2018): 0201832, 10.1371/journal.pone.0201832. PMC6104973 30133535

[16] P. S. Petrova , N. N. Viller , M. Wong , et al., “TTI‐621 (SIRPαFc): A CD47‐Blocking Innate Immune Checkpoint Inhibitor With Broad Antitumor Activity and Minimal Erythrocyte Binding,” Clinical Cancer Research 23, no. 4 (2017): 1068–1079, 10.1158/1078-0432.CCR-16-1700. 27856600

[17] X. Liu , Y. Pu , K. Cron , et al., “CD47 Blockade Triggers T Cell–mediated Destruction of Immunogenic Tumors,” Nature Medicine 21, no. 10 (2015): 1209–1215, 10.1038/nm.3931. PMC4598283 26322579

[18] R. Advani , I. Flinn , L. Popplewell , et al., “CD47 Blockade By Hu5F9‐G4 and Rituximab in Non‐Hodgkin's Lymphoma,” New England Journal of Medicine 379 (2018): 1711–1721, 10.1056/nejmoa1807315. 30380386 PMC8058634

[19] D. A. Sallman , A. S. Asch , M. M. Al Malki , et al., “The First‐in‐Class Anti‐CD47 Antibody Magrolimab (5F9) in Combination With Azacitidine Is Effective in MDS and AML Patients: Ongoing Phase 1b Results,” Blood 2019; 134: 569, 10.1182/blood-2019-126271.

[20] L. Calvin ENHANCE: Magrolimab Trials Summary: Gilead, cited, August 27, 2024, https://www.gilead.com/‐/media/files/pdfs/other/magrolimab‐trials‐summary.pdf.

[21] T. Z. Zhuang , L. Feng , A. Tyshevich , et al. Final Results of a Phase I Trial of Evorpacept (ALX 148), Lenalidomide, Rituximab for Patients with B‐cell non‐Hodgkin Lymphoma: ALX Oncology, cited, July 10, 2025, https://alxoncology.com/wp‐content/uploads/2025/05/AACR‐2025‐Final‐Results‐from‐Phase‐1‐of‐evorpacept‐lenalidomide‐rituximab‐for‐B‐cell‐NHL.pdf. 10.1158/1078-0432.CCR-25-1826 PMC12370181 40729376

[22] M. Andrianova and A. Kuznetsov , “Logic Gates Based on DNA Aptamers,” Pharmaceuticals 13, no. 11 (2020): 417, 10.3390/ph13110417. 33238657 PMC7700249

[23] H.‐M. Chen , W. van der Touw , Y. S. Wang , et al., “Blocking Immunoinhibitory Receptor LILRB2 Reprograms Tumor‐associated Myeloid Cells and Promotes Antitumor Immunity,” Journal of Clinical Investigation 128, no. 12 (2018): 5647–5662, 10.1172/JCI97570. 30352428 PMC6264729

[24] E. Allison , S. Edirimanne , J. Matthews , and S. J. Fuller , “Breast Cancer Survival Outcomes and Tumor‐Associated Macrophage Markers: A Systematic Review and Meta‐Analysis,” Oncology and Therapy 11, no. 1 (2022): 27–48, 10.1007/s40487-022-00214-3. 36484945 PMC9935786

[25] L. Pham , K. Kyritsi , T. Zhou , et al., “The Functional Roles of Immune Cells in Primary Liver Cancer,” The American Journal of Pathology 192, no. 6 (2022): 826–836, 10.1016/j.ajpath.2022.02.004. 35337836 PMC9194651

[26] G. Troiano , V. C. A. Caponio , I. Adipietro , et al., “Prognostic Significance of CD68+ and CD163+ Tumor Associated Macrophages in Head and Neck Squamous Cell Carcinoma: A Systematic Review and Meta‐analysis,” Oral Oncology 93 (2019): 66–75, 10.1016/j.oraloncology.2019.04.019. 31109698

[27] T. C. Oates , P. L. Moura , S. Cross , et al., “Defining the Proteomic Landscape of Cultured Macrophages and their Polarization Continuum,” Immunology & Cell Biology 101, no. 10 (2023): 947–963, 10.1111/imcb.12687. 37694300 PMC10953363

[28] H. Wang , J. Li , Y. Wang , et al., “IgG4‐mediated M2 Macrophage Polarization in Tertiary Lymphoid Structures of Esophageal Cancer: Implications for Immunosuppression,” Frontiers in Immunology 15 (2024): 1497783, 10.3389/fimmu.2024.1497783. 39896813 PMC11782137

[29] F. Galvez‐Cancino , A. P. Simpson , C. Costoya , et al., “Fcγ Receptors and Immunomodulatory Antibodies in Cancer,” Nature Reviews Cancer 24, no. 1 (2024): 51–71, 10.1038/s41568-023-00637-8. 38062252

[30] B.‐G. Chen , D.‐P. Xu , A. Lin , and W.‐H. Yan , “NK cytolysis is Dependent on the Proportion of HLA‐G Expression,” Human Immunology 74, no. 3 (2013): 286–289, 10.1016/j.humimm.2012.12.005. 23238216

[31] K. Kuroki , H. Matsubara , R. Kanda , et al., “Structural and Functional Basis for LILRB Immune Checkpoint Receptor Recognition of HLA‐G Isoforms,” The Journal of Immunology 203, no. 12 (2019): 3386–3394, 10.4049/jimmunol.1900562. 31694909

[32] I. Mandel , D. H. Ziv , I. Goldshtein , et al., “BND‐22, a first‐in‐class Humanized ILT2‐blocking Antibody, Promotes Antitumor Immunity and Tumor Regression,” Journal for ImmunoTherapy of Cancer 10, no. 9 2022: 004859, 10.1136/jitc-2022-004859. PMC9472153 36096532

[33] S. M. Ansell , M. B. Maris , A. M. Lesokhin , et al., “Phase I Study of the CD47 Blocker TTI‐621 in Patients With Relapsed or Refractory Hematologic Malignancies,” Clinical Cancer Research 27, no. 8 (2021): 2190–2199, 10.1158/1078-0432.CCR-20-3706. 33451977

[34] N. J. Lakhani , D. Stewart , D. L. Richardson , et al., “First‐in‐human phase I trial of the Bispecific CD47 Inhibitor and CD40 agonist Fc‐fusion Protein, SL‐172154 in Patients with Platinum‐Resistant Ovarian Cancer,” Journal for ImmunoTherapy of Cancer 13, no. 1 (2025): 010565, 10.1136/jitc-2024-010565. PMC11749819 39800375

[35] J. J. Luke , A. Saeed , B. Bashir , et al., “Phase 1 dose escalation study of DSP107, a first‐in‐class CD47 and 4‐1BB targeting multifunctional immune‐recruitment protein, in patients With advanced solid tumors,” Journal of Clinical Oncology 40 (2022): 2647–2647, 10.1200/JCO.2022.40.16_suppl.2647.

[36] Y. Pan , F. Wang , Y. Liu , J. Jiang , Y.‐G. Yang , and H. Wang , “Studying the Mechanism of CD47–SIRPα Interactions on Red Blood Cells by Single Molecule Force Spectroscopy,” Nanoscale 6, no. 17 (2014): 9951–9954, 10.1039/c4nr02889a. 25058630

[37] S. Subramanian , R. Tsai , S. Sen , K. N. Dahl , and D. E. Discher , “Membrane mobility and clustering of Integrin Associated Protein (IAP, CD47)—Major Differences between Mouse and Man and Implications for Signaling,” Blood Cells, Molecules, and Diseases 36, no. 3 (2006): 364–372, 10.1016/j.bcmd.2006.01.012. 16697668

[38] B. I. Sikic , N. Lakhani , A. Patnaik , et al., “First‐in‐Human, First‐in‐Class Phase I Trial of the Anti‐CD47 Antibody Hu5F9‐G4 in Patients With Advanced Cancers,” Journal of Clinical Oncology 37, no. 12 (2019): 946–953, 10.1200/JCO.18.02018. 30811285 PMC7186585

[39] E. M. Agency New Approach Methodologies: EU‐IN Horizon Scanning Report: EMA; 2025, cited, October 15 2025, https://www.ema.europa.eu/en/documents/report/new‐approach‐methodologies‐eu‐horizon‐scanning‐report_en.pdf.

[40] U.S. Food and Drug Administration FDA Announces Plan to Phase Out Animal Testing Requirement for Monoclonal Antibodies and Other Drugs (U.S. Food and Drug Administration 2025), https://www.fda.gov/news‐events/press‐announcements/fda‐announces‐plan‐phase‐out‐animal‐testing‐requirement‐monoclonal‐antibodies‐and‐other‐drugs.

[41] R. K. Harrison , “Phase II and phase III failures: 2013–2015,” Nature Reviews Drug Discovery 15, no. 12 (2016): 817–818, 10.1038/nrd.2016.184. 27811931

[42] M. Beilmann , K. Adkins , H. C. M. Boonen , et al., “Application of New Approach Methodologies for Nonclinical Safety Assessment of Drug Candidates,” Nature Reviews Drug Discovery 24, no. 9 (2025): 705–725, 10.1038/s41573-025-01182-9. 40316753

[43] B. Eckelman , J. Timmer , A. Razai , Q. Deveraux , K. Jones , and P. L. Nguy , “Antibodies and Methods of Use Thereof,” US9663575B2(INHIBRX LLC February 6, 2013).

[44] C. Beers , J. Corbin , D. Hodges , et al., “Anti‐hla‐g Antibodies, Compositions Comprising anti‐hla‐g Antibodies and Methods of using Anti‐hla‐g Antibodies,” US12264199B2 (Tizona Therapeutics November 18, 2021).

[45] A. M. Merchant , Z. Zhu , J. Q. Yuan , et al., “An Efficient Route to Human Bispecific IgG,” Nature Biotechnology 16, no. 7 (1998): 677–681, 10.1038/nbt0798-677. 9661204

[46] T. Schlothauer , S. Herter , C. F. Koller , et al., “Novel Human IgG1 and IgG4 Fc‐engineered Antibodies with Completely Abolished Immune Effector Functions,” Protein Engineering Design and Selection 29, no. 10 (2016): 457–466, 10.1093/protein/gzw040. 27578889

