# Supplementary material for: Selective Targeting of Immune Checkpoints HLA‐G and CD47 Using Novel Dual Signaling Protein DSP216 Promotes Innate Anticancer Immunity
Source: Adv Sci (Weinh). 2026 Feb 9;13(35):e21448. doi: 10.1002/advs.202521448 (PMC13292165; doi:10.1002/advs.202521448)
Supplement: Supplementary file 1 — Supporting File: advs74318‐sup‐0001‐SuppMat.docx. [file ADVS-13-e21448-s001.docx]

Supporting Information

Title Selective Targeting of Immune Checkpoints HLA-G and CD47 Using Novel Dual Signaling protein DSP216 Promotes Innate Anticancer Immunity

Author(s), and Corresponding Author(s)* Lisa J. Jacob, Liat Tamir, Mufeed Abdeen, Ami Tamir, Alexandra Aronin, Itai Bloch, Roy Kahn, Iris Pecker, Mark Tykocinski, Gerwin Huls, Yaron Pereg, Ayelet Chajut* and Edwin Bremer*

**Figure S1.** Representative histograms showing cells surface expression levels of HLA-G (blue histogram) and CD47 (green histogram) compared to isotype control (grey histogram) on different cell lines A) 721.221^HLA-G^ B) 721.221^EV^ C) HT1080^HLA-G^ D) HT1080^wt^ E) JEG-3 F) Binding of DSP216 to 721.221^EV^ cells (left) and 721.221^HLA-G^ cells (right) detected by AF647-IgG1 hinge Ab with increasing DSP216 concentrations from top to bottom (top histogram in each graph 0 µg/mL DSP216, bottom histogram in each graph 25 µg/mL DSP216).

**Figure S2.** Gating strategy and representative flow cytometry plots for detection of DSP216 binding to A) 721.221^HLA-G^ cells and B) 721.221^EV^ cells.

**Figure S3.** Gating strategy and representative flow cytometry plots for detection of DSP216 binding to A) HT1080^HLA-G^ cells and B) HT1080^wt^ cells.

**Figure S4.** Gating strategy and representative flow cytometry plots for detection of DSP216 binding to JEG-3 cells.

**Figure S5.** Binding of A) DSP216^wt^ and B) DSP216^V78R^ to 721.221^EV^(light blue symbols) and 721.221^HLA-G^ (dark blue symbols) cells and binding of C) DSP216^wt^ and D) DSP216^V78R^ to HT1080^wt^ (light blue symbols) and HT1080^HLA-G^ (dark blue symbols) cells detected by flow cytometry using APC-IgG1 hinge Ab (n=1).

**Figure S6.** Representative histograms of SIRPα His and LILIRB2 His binding to different cells lines A) Binding of SIRPα His to 721.221^EV^ cells (dark grey) was not blocked by DSP216 (blue). The histogram in light grey depicts detection antibody (Penta-His Ab) only. B) Binding of SIRPα His to 721.221^HLA-G^ cells (dark grey) was almost completely blocked by DSP216 (blue). The histogram in light grey depicts detection antibody (Penta-His Ab) only. C) Strong binding of LILIRB2 His to 721.221^HLA-G^ cells (cyan) was not detectable with Penta-His Ab (dark grey).

**Figure S7.** Gating strategy for detection of AF-647-DSP216 binding to mix of red blood cells (RBCs) PBMCs and HT1080.HLA-G cells

**Figure S8.** A) Flow cytometry plots AF647-DSP216 binding to CD45+ (PBMCs), CD45- (RBCs) and FITC+ (HT1080HLA-G) cells. 0 ug/mL AF647-DSP216 (grey) vs. 12.5 ug/mL AF 647-DSP216 (blue). B) AF647-DSP216 binding (12.5 µg/mL) to HT1080^HLA-G^ cells, CD45^+^ cells (PBMCs), CD45^+^/CD14^+^ (monocytes), CD45^+^/CD3^+^ (T cells), CD45^+^/CD56^+^ (NK cells) and RBCs (red blood cells)

**Figure S9.** A) Gating strategy and B) flow cytometry plots for polarization experiments. B) Exemplary histograms CD163/CD14 expression in CD11+ cells (macrophages) co-cultured

with HT1080.HLA-G cells with 0 ug/mL (grey) vs. 10 ug/mL DSP216 (blue)

**Figure S10.** DSP216 mediated phagocytosis of CellTrace™ Violet (CTV) stained cancer cells. Percentage of APC-CD11b Ab labelled macrophages positive for CTV dye is given as phagocytosis M2c+CTV [%]. Values are depicted as mean + standard deviation. A) M2c phagocytosis of HLA-G^-^ CD47^+^ 721.221^EV^ (light blue symbols, n=7, biological replicates, mean + standard deviation) cells and HLA-G^+^ CD47^+^ 721.221^HLA-G^ (dark blue symbols, n=8, biological replicates) and JEG-3 (white symbols, n=3, biological replicates) cells with increasing concentrations of DSP216 with inactive Fc (DSP216i) B) M2c phagocytosis of HLA-G^-^ CD47^+^ 721.221^EV^ (light grey symbols, n=6, biological replicates) cells and HLA-G^+^ CD47^+^ 721.221^HLA-G^ (dark grey symbols, n=6, biological replicates) and JEG-3 (white symbols, n=3, biological replicates) cells with increasing concentrations of DSP216 with active Fc (DSP216a). C) Macrophage phagocytosis of 721.221^EV^ with increasing concentrations of DSP216i (dark blue symbols, n=7, biological replicates) or DSP216a (grey symbols, n=6, biological replicates). Parametric unpaired t-test was used to test if differences in phagocytosis between treatments were significant (medium p=0.4152 ns, 2.5 µg/mL p=0.4155 ns, 5 µg/mL p=0.5728 ns, 10 µg/mL p=0.5672 ns). D) Macrophage phagocytosis of 721.221^HLA-G^ with increasing concentrations of DSP216i (dark blue symbols, n=7, biological replicates) or DSP216a (grey symbols, n=6, biological replicates). Parametric unpaired t-test was used to test if differences in phagocytosis between treatments were significant (medium p=0.3984 ns, 2.5 µg/mL p=0.1653 ns, 5 µg/mL p=0.1112 ns, 10 µg/mL p=0.4395 ns). E) Macrophage phagocytosis of JEG-3 (n=3, biological replicates) with increasing concentrations of DSP216i (dark blue symbols) or DSP216a (grey symbols). Parametric paired t-test was used to test if differences in phagocytosis between treatments were significant (2.5 µg/mL p=0.1211 ns, 5 µg/mL p=0.1530 ns, 10 µg/mL p=0.3121 ns)

**Figure S11.** Gating strategy for phagocytosis experiments

**Figure S12.** Exemplary flow cytometry plots phagocytosis experiments

**Figure S13.** Gating strategy for NK cytotoxicity experiments

**Figure S14.** Exemplary flow cytometry plots NK cytotoxicity experiments

**Table S1.** Substitutions in the DSP216 variants

| DSP216 Variants | V11 | V12 | V13 | V14 | V15 | V16 | V17 | V18 | V19 | V20 | V5 |
| --- | --- | --- | --- | --- | --- | --- | --- | --- | --- | --- | --- |
| Substitutions in LILIRB2 | T71F | V78R | S66Q | I70K | S66Q, I70K | S66Q, V78R | S66R, I70F | S66Y, T71K | S66Y, T71N | S66N, T71R | Wild type |

**Table S2.** ((Summary of binding of DSP216 variants to HT1080^HLA-G^ cells))

| DSP216 Variants/  Conc. | V11 | V12 | V13 | V14 | V15 | V16 | V17 | V18 | V19 | V20 | V5 |
| --- | --- | --- | --- | --- | --- | --- | --- | --- | --- | --- | --- |
| 20 [µg/mL]^a)^ | 203 | 353 | 123 | 129 | 151 | 177 | 161 | 155 | 187 | 149 | 100 |
| 10 [µg/mL] ^a)^ | 169 | 245 | 98 | 114 | 131 | 167 | 140 | 135 | 148 | 136 | 100 |
| 5  [µg/mL] ^a)^ | 172 | 175 | 72 | 95 | 125 | 161 | 150 | 147 | 138 | 120 | 100 |
| HLA-G Blocking^b)^ | 10-20% | 58-76% | 25-40% | 34-43% | 53% | 57% | 4-12% | 12-30% | 41-61% | 14-44% | 18-54% |

^a)^ the % binding of the respective variant compared to DSP216wt (set at 100%)

^b)^ the % of residual binding of the respective variant with pre-incubation of cells with 10 µg/mL HLA-G blocking antibody

**Document S1.** AA sequences of heteromonomers for production DSP216 variants

**Sequence 1** **LILRB2-WT-linker-IgG4 hole Fc**

LILRB2 wt (AA 22-419), linker (GGGGS)x3, Fc IgG4 (AA 99-327) hole

QTGTIPKPTLWAEPDSVITQGSPVTLSCQGSLEAQEYRLYREKKSASWITRIRPELVKNGQFHIPSITWEHTGRYGCQYYSRARWSELSDPLVLVMTGAYPKPTLSAQPSPVVTSGGRVTLQCESQVAFGGFILCKEGEEEHPQCLNSQPHARGSSRAIFSVGPVSPNRRWSHRCYGYDLNSPYVWSSPSDLLELLVPGVSKKPSLSVQPGPVVAPGESLTLQCVSDVGYDRFVLYKEGERDLRQLPGRQPQAGLSQANFTLGPVSRSYGGQYRCYGAHNLSSECSAPSDPLDILITGQIRGTPFISVQPGPTVASGENVTLLCQSWRQFHTFLLTKAGAADAPLRLRSIHEYPKYQAEFPMSPVTSAHAGTYRCYGSLNSDPYLLSHPSEPLELVVSGGGGSGGGGSGGGGSESKYGPPCPPCPAPEFEGGPSVFLFPPKPKDTLMISRTPEVTCVVVDVSQEDPEVQFNWYVDGVEVHNAKTKPREEQFNSTYRVVSVLTVLHQDWLNGKEYKCKVSNKGLPSSIEKTISKAKGQPREPQVYTLPPSQCEMTKNQVSLSCAVKGFYPSDIAVEWESNGQPENNYKTTPPVLDSDGSFFLVSRLTVDKSRWQEGNVFSCSVMHEALHNHYTQKSLSLSLGK

**Sequence 2 LILRB2-WT-linker-IgG1 hole Fc**

LILRB2 wt (AA 22-419), linker (GGGGS)x3, Fc (AA 99-330) hole

QTGTIPKPTLWAEPDSVITQGSPVTLSCQGSLEAQEYRLYREKKSASWITRIRPELVKNGQFHIPSITWEHTGRYGCQYYSRARWSELSDPLVLVMTGAYPKPTLSAQPSPVVTSGGRVTLQCESQVAFGGFILCKEGEEEHPQCLNSQPHARGSSRAIFSVGPVSPNRRWSHRCYGYDLNSPYVWSSPSDLLELLVPGVSKKPSLSVQPGPVVAPGESLTLQCVSDVGYDRFVLYKEGERDLRQLPGRQPQAGLSQANFTLGPVSRSYGGQYRCYGAHNLSSECSAPSDPLDILITGQIRGTPFISVQPGPTVASGENVTLLCQSWRQFHTFLLTKAGAADAPLRLRSIHEYPKYQAEFPMSPVTSAHAGTYRCYGSLNSDPYLLSHPSEPLELVVSGGGGSGGGGSGGGGSEPKSSDKTHTCPPCPAPELLGGPSVFLFPPKPKDTLMISRTPEVTCVVVDVSHEDPEVKFNWYVDGVEVHNAKTKPREEQYNSTYRVVSVLTVLHQDWLNGKEYKCKVSNKALPAPIEKTISKAKGQPREPQVCTLPPSRDELTKNQVSLSCAVKGFYPSDIAVEWESNGQPENNYKTTPPVLDSDGSFFLVSKLTVDKSRWQQGNVFSCSVMHEALHNHYTQKSLSLSPGK

**Sequence 3 LILRB2-V78R-linker-IgG1 hole Fc**

LILRB2 V78R (AA 22-419), linker (GGGGS)x3, Fc (AA 99-330) hole

QTGTIPKPTLWAEPDSVITQGSPVTLSCQGSLEAQEYRLYREKKSASWITRIRPEL**R**KNGQFHIPSITWEHTGRYGCQYYSRARWSELSDPLVLVMTGAYPKPTLSAQPSPVVTSGGRVTLQCESQVAFGGFILCKEGEEEHPQCLNSQPHARGSSRAIFSVGPVSPNRRWSHRCYGYDLNSPYVWSSPSDLLELLVPGVSKKPSLSVQPGPVVAPGESLTLQCVSDVGYDRFVLYKEGERDLRQLPGRQPQAGLSQANFTLGPVSRSYGGQYRCYGAHNLSSECSAPSDPLDILITGQIRGTPFISVQPGPTVASGENVTLLCQSWRQFHTFLLTKAGAADAPLRLRSIHEYPKYQAEFPMSPVTSAHAGTYRCYGSLNSDPYLLSHPSEPLELVVSGGGGSGGGGSGGGGSEPKSSDKTHTCPPCPAPELLGGPSVFLFPPKPKDTLMISRTPEVTCVVVDVSHEDPEVKFNWYVDGVEVHNAKTKPREEQYNSTYRVVSVLTVLHQDWLNGKEYKCKVSNKALPAPIEKTISKAKGQPREPQVCTLPPSRDELTKNQVSLSCAVKGFYPSDIAVEWESNGQPENNYKTTPPVLDSDGSFFLVSKLTVDKSRWQQGNVFSCSVMHEALHNHYTQKSLSLSPGK

**Sequence 4** **SIRPα-linker-IgG4 knob Fc**

SIRPα (AA 31-373,) linker (GGGGS)x2, Fc IgG4 (AA 99-327) knob

EEELQVIQPDKSVLVAAGETATLRCTATSLIPVGPIQWFRGAGPGRELIYNQKEGHFPRVTTVSDLTKRNNMDFSIRIGNITPADAGTYYCVKFRKGSPDDVEFKSGAGTELSVRAKPSAPVVSGPAARATPQHTVSFTCESHGFSPRDITLKWFKNGNELSDFQTNVDPVGESVSYSIHSTAKVVLTREDVHSQVICEVAHVTLQGDPLRGTANLSETIRVPPTLEVTQQPVRAENQVNVTCQVRKFYPQRLQLTWLENGNVSRTETASTVTENKDGTYNWMSWLLVNVSAHRDDVKLTCQVEHDGQPAVSKSHDLKVSAHPKEQGSNTAAENTGSNERNIYGGGGSGGGGSESKYGPPCPPCPAPEFEGGPSVFLFPPKPKDTLMISRTPEVTCVVVDVSQEDPEVQFNWYVDGVEVHNAKTKPREEQFNSTYRVVSVLTVLHQDWLNGKEYKCKVSNKGLPSSIEKTISKAKGQPREPQVCTLPPSQEEMTKNQVSLWCLVKGFYPSDIAVEWESNGQPENNYKTTPPVLDSDGSFFLYSRLTVDKSRWQEGNVFSCSVMHEALHNHYTQKSLSLSLGK

**Sequence 5** **SIRPα-linker-IgG1 knob Fc,**

SIRPα (AA 31-373,) linker (GGGGS)x2, Fc IgG1 (AA 99-330) knob

EEELQVIQPDKSVLVAAGETATLRCTATSLIPVGPIQWFRGAGPGRELIYNQKEGHFPRVTTVSDLTKRNNMDFSIRIGNITPADAGTYYCVKFRKGSPDDVEFKSGAGTELSVRAKPSAPVVSGPAARATPQHTVSFTCESHGFSPRDITLKWFKNGNELSDFQTNVDPVGESVSYSIHSTAKVVLTREDVHSQVICEVAHVTLQGDPLRGTANLSETIRVPPTLEVTQQPVRAENQVNVTCQVRKFYPQRLQLTWLENGNVSRTETASTVTENKDGTYNWMSWLLVNVSAHRDDVKLTCQVEHDGQPAVSKSHDLKVSAHPKEQGSNTAAENTGSNERNIYGGGGSGGGGSEPKSSDKTHTCPPCPAPELLGGPSVFLFPPKPKDTLMISRTPEVTCVVVDVSHEDPEVKFNWYVDGVEVHNAKTKPREEQYNSTYRVVSVLTVLHQDWLNGKEYKCKVSNKALPAPIEKTISKAKGQPREPQVYTLPPCRDELTKNQVSLWCLVKGFYPSDIAVEWESNGQPENNYKTTPPVLDSDGSFFLYSKLTVDKSRWQQGNVFSCSVMHEALHNHYTQKSLSLSPGK

**Sequence 6 LILRB2-WT-linker-IgG1 hole Fc LALA**

LILRB2 wt (AA 22-419), linker (GGGGS)x3, Fc (AA 99-330) hole LALA

QTGTIPKPTLWAEPDSVITQGSPVTLSCQGSLEAQEYRLYREKKSASWITRIRPELVKNGQFHIPSITWEHTGRYGCQYYSRARWSELSDPLVLVMTGAYPKPTLSAQPSPVVTSGGRVTLQCESQVAFGGFILCKEGEEEHPQCLNSQPHARGSSRAIFSVGPVSPNRRWSHRCYGYDLNSPYVWSSPSDLLELLVPGVSKKPSLSVQPGPVVAPGESLTLQCVSDVGYDRFVLYKEGERDLRQLPGRQPQAGLSQANFTLGPVSRSYGGQYRCYGAHNLSSECSAPSDPLDILITGQIRGTPFISVQPGPTVASGENVTLLCQSWRQFHTFLLTKAGAADAPLRLRSIHEYPKYQAEFPMSPVTSAHAGTYRCYGSLNSDPYLLSHPSEPLELVVSGGGGSGGGGSGGGGSEPKSSDKTHTCPPCPAPE**AA**GGPSVFLFPPKPKDTLMISRTPEVTCVVVDVSHEDPEVKFNWYVDGVEVHNAKTKPREEQYNSTYRVVSVLTVLHQDWLNGKEYKCKVSNKALPAPIEKTISKAKGQPREPQVCTLPPSRDELTKNQVSLSCAVKGFYPSDIAVEWESNGQPENNYKTTPPVLDSDGSFFLVSKLTVDKSRWQQGNVFSCSVMHEALHNHYTQKSLSLSPGK

**Sequence 7 LILRB2-V78R-linker-IgG1 hole Fc LALA**

LILRB2 V78R (AA 22-419), linker (GGGGS)x3, Fc (AA 99-330) hole LALA

QTGTIPKPTLWAEPDSVITQGSPVTLSCQGSLEAQEYRLYREKKSASWITRIRPEL**R**KNGQFHIPSITWEHTGRYGCQYYSRARWSELSDPLVLVMTGAYPKPTLSAQPSPVVTSGGRVTLQCESQVAFGGFILCKEGEEEHPQCLNSQPHARGSSRAIFSVGPVSPNRRWSHRCYGYDLNSPYVWSSPSDLLELLVPGVSKKPSLSVQPGPVVAPGESLTLQCVSDVGYDRFVLYKEGERDLRQLPGRQPQAGLSQANFTLGPVSRSYGGQYRCYGAHNLSSECSAPSDPLDILITGQIRGTPFISVQPGPTVASGENVTLLCQSWRQFHTFLLTKAGAADAPLRLRSIHEYPKYQAEFPMSPVTSAHAGTYRCYGSLNSDPYLLSHPSEPLELVVSGGGGSGGGGSGGGGSEPKSSDKTHTCPPCPAPE**AA**GGPSVFLFPPKPKDTLMISRTPEVTCVVVDVSHEDPEVKFNWYVDGVEVHNAKTKPREEQYNSTYRVVSVLTVLHQDWLNGKEYKCKVSNKALPAPIEKTISKAKGQPREPQVCTLPPSRDELTKNQVSLSCAVKGFYPSDIAVEWESNGQPENNYKTTPPVLDSDGSFFLVSKLTVDKSRWQQGNVFSCSVMHEALHNHYTQKSLSLSPGK

**Sequence 8 SIRPα-linker-IgG1 knob Fc LALA**

SIRPα (AA 31-373,) linker (GGGGS)x2, Fc IgG1 (AA 99-330) knob LALA

EEELQVIQPDKSVLVAAGETATLRCTATSLIPVGPIQWFRGAGPGRELIYNQKEGHFPRVTTVSDLTKRNNMDFSIRIGNITPADAGTYYCVKFRKGSPDDVEFKSGAGTELSVRAKPSAPVVSGPAARATPQHTVSFTCESHGFSPRDITLKWFKNGNELSDFQTNVDPVGESVSYSIHSTAKVVLTREDVHSQVICEVAHVTLQGDPLRGTANLSETIRVPPTLEVTQQPVRAENQVNVTCQVRKFYPQRLQLTWLENGNVSRTETASTVTENKDGTYNWMSWLLVNVSAHRDDVKLTCQVEHDGQPAVSKSHDLKVSAHPKEQGSNTAAENTGSNERNIYGGGGSGGGGSEPKSSDKTHTCPPCPAPE**AA**GGPSVFLFPPKPKDTLMISRTPEVTCVVVDVSHEDPEVKFNWYVDGVEVHNAKTKPREEQYNSTYRVVSVLTVLHQDWLNGKEYKCKVSNKALPAPIEKTISKAKGQPREPQVYTLPPCRDELTKNQVSLWCLVKGFYPSDIAVEWESNGQPENNYKTTPPVLDSDGSFFLYSKLTVDKSRWQQGNVFSCSVMHEALHNHYTQKSLSLSPGK

**Document S2.** AA sequences of CD47-Fc

HAQLLFNKTKSVEFTFCNDTVVIPCFVTNMEAQNTTEVYVKWKFKGRDIYTFDGALNKSTVPTDFSSAKIEVSQLLKGDASLKMDKSDAVSHTGNYTCEVTELTREGETIIELKYRVVSWFSPEPKSSDKTHTCPPCPAPEAEGAPSVFLFPPKPKDTLMISRTPEVTCVVVDVSHEDPEVKFNWYVDGVEVHNAKTKPREEQYNSTYRVVSVLTVLHQDWLNGKEYKCKVSNKALPAPIEKTISKAKGQPREPQVYTLPPSRDELTKNQVSLTCLVKGFYPSDIAVEWESNGQPENNYKATPPVLDSDGSFFLYSKLTVDKSRWQQGNVFSCSVMHEALHNHYTQKSLSLSPGK.
